# Modelling the key drivers of an aerial *Phytophthora* foliar disease epidemic, from the needles to the whole plant

**DOI:** 10.1371/journal.pone.0216161

**Published:** 2019-05-28

**Authors:** Mireia Gomez-Gallego, Ralf Gommers, Martin Karl-Friedrich Bader, Nari Michelle Williams

**Affiliations:** 1 Institute for Applied Ecology New Zealand, School of Sciences, Auckland University of Technology, Auckland, New Zealand; 2 New Zealand Forest Research Institute (Scion), Rotorua, New Zealand; University of Nebraska-Lincoln, UNITED STATES

## Abstract

Understanding the epidemiology of infectious diseases in a host population is a major challenge in forestry. Radiata pine plantations in New Zealand are impacted by a foliar disease, red needle cast (RNC), caused by *Phytophthora pluvialis*. This pathogen is dispersed by water splash with polycyclic infection affecting the lower part of the tree canopy. In this study, we extended an SI (Susceptible-Infectious) model presented for RNC to analyse the key epidemiological drivers. We conducted two experiments to empirically fit the extended model: a detached-needle assay and an *in vivo* inoculation. We used the detached-needle assay data to compare resistant and susceptible genotypes, and the *in vivo* inoculation data was used to inform sustained infection of the whole plant. We also compared isolations and real-time quantitative PCR (qPCR) to assess *P*. *pluvialis* infection. The primary infection rate and the incubation time were similar for susceptible and resistant genotypes. The pathogen death rate was 2.5 times higher for resistant than susceptible genotypes. Further, external proliferation of mycelium and sporangia were only observed on 28% of the resistant ramets compared to 90% of the susceptible ones. Detection methods were the single most important factor influencing parameter estimates of the model, giving qualitatively different epidemic outputs. In the early stages of infection, qPCR proved to be more efficient than isolations but the reverse was true at later points in time. Isolations were not influenced by the presence of lesions in the needles, while 19% of lesioned needle maximized qPCR detection. A primary infection peak identified via qPCR occurred at 4 days after inoculation (dai) with a secondary peak observed 22 dai. Our results have important implications to the management of RNC, by highlighting the main differences in the response of susceptible and resistant genotypes, and comparing the most common assessment methods to detect RNC epidemics.

## Introduction

Research on the epidemiology of plant pathogens and the development of sustainable strategies for disease control might have never been so pressing. The frequency of the emergence of invasive plant-infecting fungi and fungal-like organisms has increased enormously with international trade in recent decades [1]. Forestry plantations have been threatened by such invasive diseases with new associations between pathogenic species and non-native hosts being increasingly noted globally [2–4]. The efficacy of sustainable disease management in plantation forests is underpinned by the fundamental dynamics of disease epidemiology, interactions with susceptible hosts, the biological limitations and potential of the pathogen, and the respective physiologies within a mutual environment. Understanding this dynamic interaction presents an opportunity to develop smarter disease management strategies and reduce the impact of disease in the field.

*Phytophthora* species are a relatively newly recognized cause of foliar diseases in *Pinus radiata D*. *Don*, the key plantation forest species in New Zealand (NZ), Chile and Australia [5–7]. New reports of ‘Daño Foliar de Pino’ in Chile in 2008, and observations of the red needle cast (RNC) disease in New Zealand soon thereafter, highlighted the potential significance of foliar *Phytophthora* impacts on *P*. *radiata* plantation forestry [8,9]. RNC was first reported in *P*. *radiata* stands on the east coast of New Zealand in late April 2008 [9]. Symptoms of RNC emerge in late autumn and continue to develop through until mid to late spring. Infection is characterised by an olive discoloration of affected needles, frequently accompanied by resinous dark bands on the needles [9]. Affected foliage quickly turns orange to brown, lending a reddish hue to the affected crown. Infected needle fascicles are cast with new needles emerging in late spring often unaffected by disease. Initial studies have shown that severe defoliation events associated with RNC can reduce tree growth by up to 38% in the year following infection [10]. Isolations from symptomatic needles onto *Phytophthora* selective media yielded the newly described *Phytophthora pluvialis* Reeser, Sutton and Hansen. The same pathogen was subsequently identified as a contributing pathogen of early defoliation in Douglas-fir in NZ and the US Pacific Northwest [9,11].

Early work on the RNC disease focused on confirming the cause, disease expression, timing and risks for trade. However, limited work has been done to date to progress a deeper understanding of the epidemiology of the disease. Some knowledge gaps still remain on the drivers of sustained RNC infection, characterized by the polycyclic nature of the splash-dispersed *P*. *pluvialis*, on the timing and peaks of primary and secondary infections, and on host susceptibility and resistance to RNC. Further, several diagnostic methods have been used to date, i.e. isolations in culture media and real-time quantitative PCR (qPCR) [12,13], but no comprehensive analysis on their efficiency and potential shortcomings have been performed.

Epidemiological models have been previously applied to give insight into key aspects of disease management. For example, compartmental models have explored disease prediction through the characterization of latent infectious periods and transmission rates [14–17], while others have focused on the efficacy of control methods, such as cultural practices [18], or induced resistance [19]. In this paper, we further explore a generic mathematical SI (susceptible-infectious) model for RNC that articulates the RNC disease dynamic introduced by Wake et al [20]. Our contributions are twofold. First, we extend the SI model to account for the dynamics of pathogen-host interaction. Then, we apply the extended model to two experiments: a detached-needle assay and an *in vivo* inoculation. The empirical data from those two experiments allowed us to (1) determine incubation times, infection rates, and pathogen mortality rates for susceptible and resistant genotypes. Moreover, we (2) quantified differences between the main RNC diagnostic methods (isolation and qPCR), and (3) analysed primary and secondary infection peaks in a sustained RNC infection episode. For our first aim, we performed a detached-needle assay to collect empirical data to fit the model. This assay also provided data on the latent infectious periods, and the peak of primary infection. For our second and third aims, an *in vivo* inoculation experiment was carried out in a controlled temperature fog room. This data was further analysed to evaluate the correlations between presence of lesions and the two main diagnostic methods, isolations and qPCR detection. This paper provides the first investigation into the epidemiology of the RNC disease.

## Material and methods

We performed two different inoculation experiments in order to parameterise an extended version of an established model for red needle cast [20]. To characterize the primary infection of the pathogen, i.e. the infection initiated from incoming inoculum [21], we designed a detached-needle assay, where only individual detached pine needles were inoculated and monitored for 11 days. This period has been previously demonstrated to cover the period of primary infection up to peak infection and to needle senescence [22]. To study the secondary infection by the pathogen, i.e. the infection originated by spread from infected to susceptible needles [21], we performed an *in vivo* inoculation experiment. Whole plants were inoculated with the pathogen and monitored for a longer period, up to 28 days, within which secondary infection could be observed. Whole plants provide sufficient needle mass to host secondary infection events.

### Inoculum preparation

Zoospore inoculum was prepared as described previously by Rolando et al. [23] with modification to produce sufficient inoculum for the scale of the trials. *P*. *pluvialis* isolates used for inoculum preparation for the detached-needle assay were NZFS3901, 3902, 4014, 4015, 4016 and 4017, and NZFS4019, 4234, 4267, 4268, 4317, 4325, for the *in vivo* inoculation. Different isolates were used as the pathogenicity phenotype of *P*. *pluvialis* has been found to attenuate over time with laboratory culturing. All isolates were routinely passed through pine needles prior to each experiment to confirm their ability to infect. A mix of isolates was used to enable selection for the most virulent one [24] and favour infection, as commonly performed in epidemiological studies [25,26]. All isolates used as source of inoculum were identified using morphological and molecular techniques. Isolates have typically remained active for up to 12 months, but were routinely removed from the screening programme as soon as they failed to re-infect *P*. *radiata* and replaced with fresh isolates sourced from the field. The isolates were cultured on carrot agar at 17°C for three days. Plugs of agar and mycelium were taken from the leading edge of the colonies, flooded with 55 ml of clarified carrot broth [27] in vented 175 cm^2^ flat bottomed flasks (Nunc EasYFlasks, Thermo Scientific) and incubated for three days in the dark at 17°C. The resulting mycelial mats were rinsed thoroughly in a steady stream of deionized water, drained, and then reflooded with 55 ml of sterile pond water. The pond water was collected from a local pond and autoclaved before being used to induce sporangia production and sporulation of *P*. *pluvialis* cultures. The flasks were re-incubated in the dark for further three days. Zoospore release was induced by cold-shocking the cultures at 4°C in the dark for 45 min, followed by exposure at room temperature (21 ºC), and by placing cultures on a light box if required. Upon release, the zoospore inoculum of all six isolates was combined to form a single mixed inoculum. Zoospore concentrations were determined using a haemocytometer, reaching a minimum of 1 x 10^4^ zoospores/ml, for the detached-needle assay, and 3 x 10^3^ zoospores/ml, for the whole-plant assay. Zoospore solutions were applied within two hours of preparation.

### Detached-needle assay

#### Plant material and inoculation treatment

*Pinus radiata* genotypes were preselected from the Radiata Pine Breeding Company’s Elite Clones series as published previously [22]. Four genotypes were selected for higher susceptibility and four genotypes for higher resistance to RNC, respectively. Plants were clonally propagated from stool beds as bare-rooted cuttings, planted in Scion’s nursery in Rotorua, New Zealand. Thirty-five healthy fascicles were collected from each of the six ramets (clonal plants) from each of the eight genotypes. The fascicles were assigned to treatments following a split-split plot design with two independent blocks, water control and *P*. *pluvialis* inoculation (whole plots), seven sampling time points (0, 1, 3, 5, 7, 9, 11 days after inoculation) separated in single incubation trays (split plot), and eight genotypes (split-split plot). Five fascicles from each ramet and each genotype were place in each tray, after their exposure to dip inoculation in 15-mL flat bottom plastic tubes containing 4.5 mL of either water or *P*. *pluvialis* zoospore suspension. The experimental design is shown in S1 Fig, only for control treatment, which is identical to *P*. *pluvialis* inoculation treatment.

Each tube accommodated five fascicles and was inoculated with 4.5 ml of either the *P*. *pluvialis* zoospore suspension (see ‘Inoculum preparation’) or sterile pond water (control) and incubated at room temperature overnight (18 hours). Fascicles were placed on trays moistened with wet paper towels, covered in plastic film and incubated in a controlled environment (17°C, 65–70% relative humidity, 14 h photoperiod) for 10 days. The allocation of individual samples within trays was randomized at the block level and the position of incubation of each tray within the growth room randomized to account for variation within the incubation room. Tubes were randomized prior to inoculation with the layout carried through for needle incubation.

#### Assessment of P. pluvialis infection

Pathogen presence and growth was assessed by measuring lesion length, pathogen isolation in culture media, and by fluorescence microscopy observations. At each harvest point, the needles within each fascicle were separated, and the lesions counted and measured to the nearest mm. The five fascicles within each replicate were allocated at random for parallel analysis by isolation, immersed in FAA fixative (50% ethanol, 5% (v/v) acetic acid, 3.7% (v/v) formaldehyde), snap frozen and stored at -80°C for further analyses.

Total needle length and lesion length, characteristic of early symptoms of RNC (olive-coloured discoloration and black bands, [9]) were recorded for each needle, the percentage of lesioned needle length calculated, and averaged for each fascicle. For isolations, the proximal 3 cm of each needle that had been exposed to inoculum/water was surface sterilised for 30 seconds in 70% ethanol, rinsed in sterile deionised water and patted dry with paper towels. Each needle was sectioned into three pieces and plated onto *Phytophthora* selective CRN media (10% carrot broth, 15 g/L agar, 200 mg/L ampicillin, 0.05 g/L nystatin, 0.01 g/L rifampicin, 0.4 ml/L pimaricin). Plates were incubated at 17°C in the dark for up to 14 days and the proportion of isolations recorded for each fascicle. *Phytophthora pluvialis* colonies were morphologically identified [28] by subculturing and inducing sporangia production in pond water [27].

Microscopic observations were made of the production of *P*. *pluvialis* spore and mycelial structures on the surface of pine needles fixed at harvest and stored in FAA fixative. Whole fascicles were removed from the FAA solution, patted dry, the proximal 3 cm dissected and placed in a 0.01% solution of calcofluor white dissolved in ultra-pure water (Sigma Aldrich) for 30 seconds. Excess calcofluor was removed by briefly rinsing in sterile deionized water and the sections dried on a tissue before placing on a microscope slide without a cover slip. Needles were observed using an Olympus BX61 fluorescence microscope under UV excitation with the presence of *P*. *pluvialis* mycelium and sporangia noted for each needle within fascicle.

### *In vivo* inoculation

#### Plant material and inoculation treatment

The experiment was conducted on 3-year-old *P*. *radiata* grafts (Proseed, Scion nursery) of four RNC-susceptible genotypes. Ninety grafts were assigned to treatments following a split-plot design with two independent blocks, water control and *P*. *pluvialis* inoculation (whole plots), and four genotypes (split plot). The experimental design is shown in S2 Fig.

The experiment was performed in two fog rooms with an overhead misting system under identical conditions: 10 h photoperiod, 95–100% relative humidity, 10°C at night/ 17°C during the day. Mist was applied for 1 min continuously, every 10 min. At the beginning of the experiment, needles and branches of the grafts already placed in the fog room, as per *P*. *pluvialis* inoculation treatment, were thoroughly sprayed with zoospore suspensions (see ‘Inoculum preparation’) using a low-pressure spray (1 MPa). Control grafts were placed on the control fog room without any inoculation treatment, and kept under the same conditions.

#### Assessment of P. pluvialis infection

*P*. *pluvialis* infection was monitored by performing isolations and qPCR. Two fascicles (two to four needles per fascicle) per plant were harvested three times a week from four days after inoculation (dai) for one month totalling 12 sampling dates. Fascicles were kept in dampened paper towels and processed within 4 hours of collection. Total needle length and lesion length were recorded, and the percentage of lesioned needle area calculated. One fascicle was surface sterilized in 70% ethanol for 30 s, rinsed in sterile deionized water, blotted dry on filter paper, and plated onto *Phytophthora* selective CRN media (see above). *Phytophthora pluvialis* colonies were identified morphologically [28] by subculturing in 10% carrot agar and inducing sporangia production in pond water [27]. Success or failure in recovering *P*. *pluvialis* was recorded for each fascicle.

The second fascicle was immediately frozen with liquid nitrogen in 5 ml tubes. Genomic DNA extractions and qPCR were performed by a commercial service provider (Slipstream Automation, Palmerston North, New Zealand) using a CTAB/Chloroform-based procedure in an automated robotic workflow. qPCR reactions targeting *P*. *radiata* and *P*. *pluvialis* were performed separately (uniplex reactions) in 10-μl aliquots with 1X TaqMan Universal Master Mix, 0.2 μM BHQ-labelled probe, 0.4 μM forward and reverse primers, 1 μl DNA template. Probe and primer sets used for each organism are listed in Table 1 [29]. The following qPCR conditions were used for *P*. *radiata*: initial incubation of 10 min at 95°C followed by incubation of 40 cycles of 10 s at 95°C and 20 s at 58°C. For *P*. *pluvialis*, reaction conditions were: initial incubation of 10 min at 95°C followed by incubation of 40 cycles of 15 s at 96°C and 1 min at 60°C.

**Table 1:** TaqMan probe, primer sets and target genes for relative quantification of *Pinus radiata* and *Phytophthora pluvialis* in DNA extracts of *P*. *radiata* needles. ^1^ TaqMan probes: *P*. *radiata* probe is labelled with the reporter CAL Fluor Orange 560 (Amidite, emission 556 nm) on the 5’ end, the reporter dye for *P*. *pluvialis* is FAM (6-carboxy-fluorescein; emission 518 nm), all probes are labelled with BHQ1 on the 3’ as a quencher; ^2^ Forward primer; ^3^ Reverse primer.

Species	Target gene	Probe/ primer	Sequence (5’ → 3’)	Product size
*P*. *radiata*^a^	*CAD*	Probe945^1^	5’ /CAL Fluor Orange 560/TGT GAA CCA TGA CGG CAC CC/BHQ1/ 3’	101 bp
		CAD918F^2^	CAG CAA GAG GAT TTG GAC CTA	
		CAD1019R^3^	TTC AAT ACC CAC ATC TGA TCA AC	
*P*. *pluvialis*^b^	*Ypt1*	Ypap^1^	5’ /FAM/TCC TCC TTG GTA ACG CTA A/BHQ1/ 3’	227 bp
		Ypap2F^2^	AAC TTG GTG CGG TAT TCA CG	
		Ypap2R^3^	ATC AGT TAG CTC CTT TCC	

^a^ [29]

^b^ McDougal et al. in prep

Standard curves were constructed amplifying DNA from the two target species: *P*. *radiata* and *P*. *pluvialis*. Total DNA was serially diluted 10-fold with sterile PCR-grade water. Linear regressions were performed between log-transformed DNA concentration and Cq using the software R version 3.5.1 (R Core Team 2017). Coefficients of determination (R^2^) obtained from linear regression for both species were 1. Standard curves were used to approximate the concentration in ng/μL for each species and sample. The ratio of *P*. *pluvialis* DNA to *P*. *radiata* DNA was used as a measure of pathogen abundance.

#### Assessment of quantification methods and symptom expression

To assess the correlation between isolation and qPCR methods with symptom expression, statistical analyses was performed using the software R version 3.5.1 (R Core Team 2017). Both *P*. *pluvialis* isolation and qPCR detection success (binary variables) were modelled using generalized additive mixed models (GAMMs) with binomial error distribution (function gamm4, package gamm4, [30]). *P*. *pluvialis* DNA abundance was likewise modelled using a tweedie error distribution (p = 1.44). In the three models, ‘plant identity’ nested within ‘genotype’ were incorporated as random effects to account for repeated measures. ‘Dai’ and ‘lesioned needle length’ (proportion) were included in the smoother term separately. Their interaction was tested using bivariate smoothers (t2 argument), followed by a model comparison based on the Akaike Information Criterion (AIC). A difference in AIC of 5 or more scores led us to opt for the model with the lower AIC value. Backward model selection using AIC comparison was further used to determine the optimal smoother terms.

### Model development

**Fig 1:**
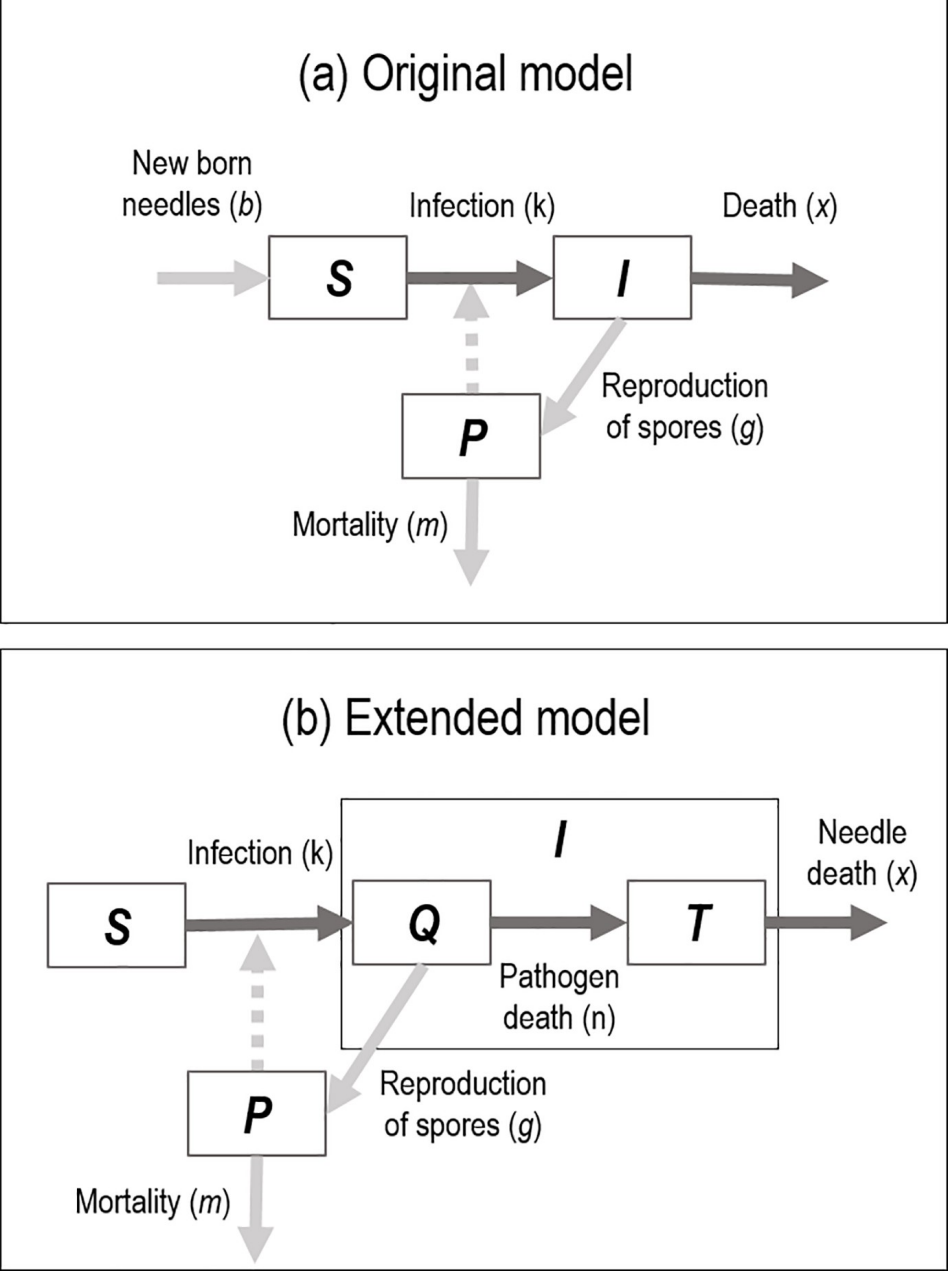
Modifications of the SI original model (a) by [20], to obtain the SIPQ model (b) to use the empirical data provided by the detached-needle assay and *in vivo* inoculation.

Wake et al [20] formulated an SI model for the RNC disease (hereafter referred to as original model, Fig 1A). The original model presents two compartmented state variables for pine needles: susceptible, but not yet infected, i.e. healthy (*S*), and infected (*I*). Infected needles cannot return to the *S* compartment as they die and cast after infection at a rate *x* (Fig 1A). The additional state variable (*P*) accounts for the pathogen spore density, i.e. the amount of pathogen units that can generate infection (Fig 1A). The pathogen inoculum (*P*) is increased by the spores produced on the infected needles (*g*), and tempered by the mortality rate of spores (*m*). We extended this model (Fig 1B, hereafter referred to as extended model or SIPQ) to account for the pathogen-host dynamics once needles become infected. To do this, we divided the *I* compartment into two new compartments: needles that are infected with live pathogen on them (*Q*, Fig 1B), and needles that are infected with non-viable pathogen on them, i.e. terminal needles (*T*). Therefore, the total proportion of infected needles (*I*) can host either infectious (*Q*) or non-viable pathogen (*T*), with *I* = *Q*+*T*. This division helps clarify the pathogen-host dynamic, with implications in the epidemiology of the disease. On the one hand, infected needles with live pathogen (*Q*) are the only infectious ones, i.e. capable of supporting reproduction of the pathogen and inoculum build-up. On the other hand, terminal needles (*T*) are not infectious anymore. Hence, they do not contribute to the inoculum build-up, but they can have an impact on the tree physiology as they are still attached to the tree. Those needles are presumably carbon sinks, due to the impairment of the photosynthetic apparatus usually associated to foliar infection [31]. This may have an impact on different other processes with feedbacks to the disease epidemic, such as the amount of available carbon and its allocation to defense, or to crown expansion (development of new born needles). Thus, the extended model allows for future incorporation of these feedbacks.

The reproduction of spores takes place in the *Q* compartment and feeds the *P* compartment, as per the original model. This extension helps differentiate between needle death and pathogen death, and allows the empirical contrast of the model fit using data from the detached-needle assay and *in vivo* inoculation. For the purpose of the present study, we omitted the emergence of new born needles input to the *S* compartment, since the conditions of the experiments performed here do not allow new needles to develop and enter the system. The detached-needle assay develops on a fixed amount of needles, and the *in vivo* inoculation experiment lasts 28 days, during which no new needles were formed, due to the winter conditions. However, it is important to note that, for multi-season experiments, new born needles would be developed, and other would naturally die. In this case, *b* (in the original model) will be different from 0, and a new flow should be added from the *S* compartment which would correspond to the needles dying for reasons other than the RNC disease.

For the SIPQ model, the five differential equations for proportion of healthy needles (*S*, Eq 1), spore density (*P*, Eq 2), proportion of needles with infectious pathogen (*Q*, Eq 3), and proportion of needles with non-viable pathogen (*T*, Eq 4) are:

$$\frac{dS}{dt}=-kSP$$ (1)

$$\frac{dP}{dt}=gI\left(1-\frac{min(P,P_{max})}{P_{max}}\right)-mP$$ (2)

$$\frac{dQ}{dt}=kSP-nQ$$ (3)

$$\frac{dT}{dt}=nQ-xT$$ (4)

**Fig 2:**
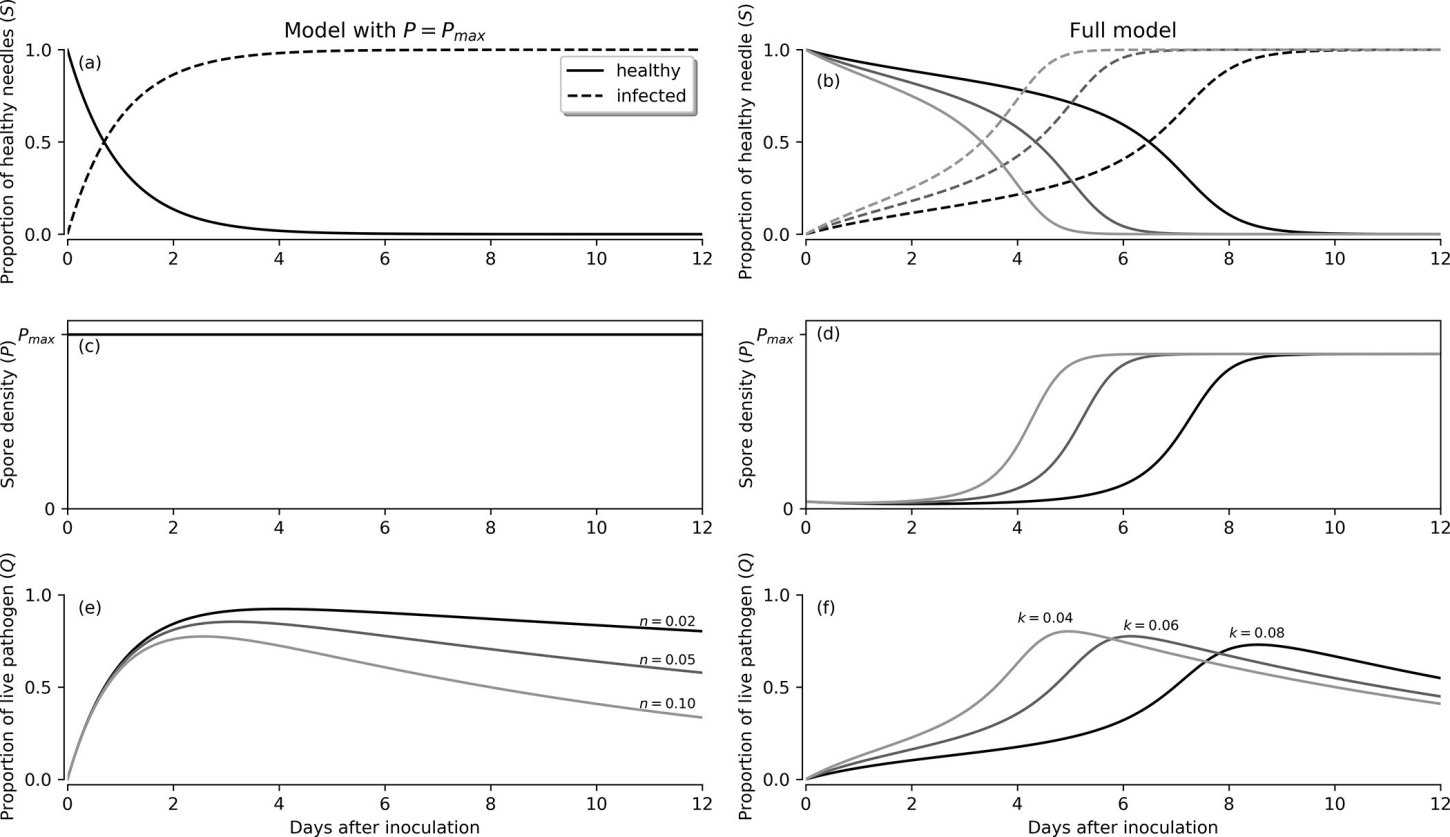
Illustration of the time-dependent behaviour of the model ODE (ordinary differential equations) of Eqs 1–2 and 4 under various conditions. First column plots correspond to the model extended for the detached-needle assay, and second column plots, to the extension for the *in vivo* inoculation. Proportion of healthy needles, *S* (a-b), spore density, *P* (c-d), and proportion of needles with live pathogen, *Q* (e-f) are shown.

As the model acts on a proportional interpretation, the total proportion of infected needles (*I*) is given by *I* = 1−*S*. Table 2 gives a description of all model parameters in Eqs 1–4. Fig 2 illustrates model behaviour with two different assumptions: *P* saturated and constant (first-column plots, see section ‘Applying the model to the detached-needle assay’), and *P* varying along the infection period (second-column plots, see section ‘Applying the model to the *in vivo* inoculation’). The effect of changes in the two most important model parameters–*k*, infection rate, and *n*, pathogen death rate–is also shown.

**Table 2:** Description of variables and parameters used in the SIPQ model for RNC infection with *Phytophthora pluvialis*.

Parameter	Description
*S*	state variable for the proportion of needles that is healthy
*I*	state variable for the proportion of needles that has been infected
*P*	state variable for spore density on the needle surface
*Q*	state variable for the proportion of needles with infectious pathogen in them
*T*	state variable for the proportion of needles with non-viable pathogen in them
*x*	death rate of infected needles
k	infection rate
g	spore birth rate
*m*	death rate of spores
n	death rate of pathogen in host material
*P*_*max*_	carrying capacity of spores for each needle

### Applying the epidemiological model

#### Applying the model to the detached-needle assay

**Fig 3:**
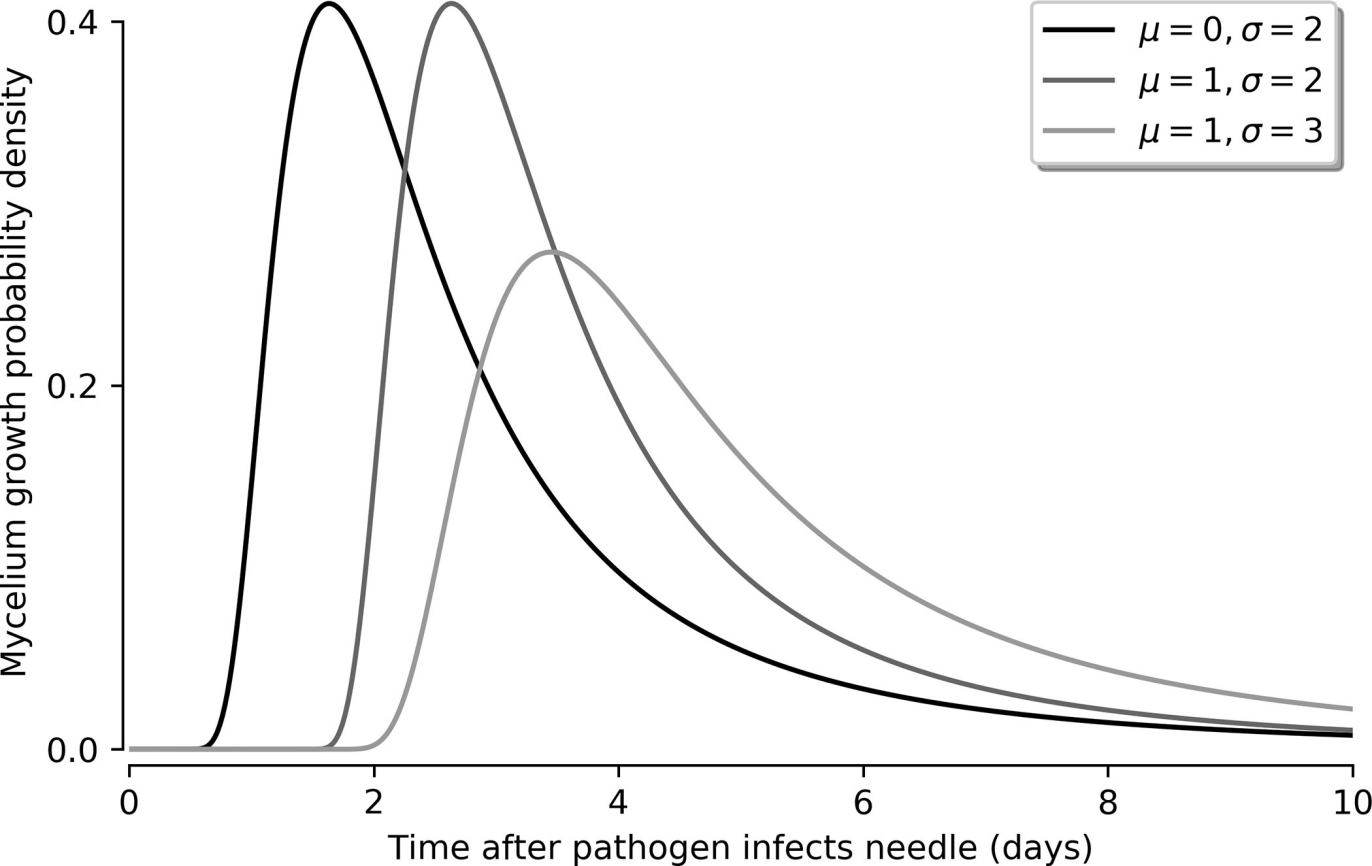
Shape of distribution for modelling incubation time (*τ*_m_) for different values of the location (μ) and scale (σ) parameters of that distribution.

In the detached-needle assay, individual fascicles were dip inoculated with a zoospore suspension. In this process, the zoospores primarily infect at the surface of the inoculum due to negative gravitaxis and oxygen chemotaxis [32,33], at which point they encyst on the plant surface, and germinate penetrating through to the intercellular space [34]. Following a period of intercellular growth, mycelium emerges through stomatal openings and sporangia are produced. We designate incubation time (*τ*_*m*_) to the time delay from inoculation to the re-emergence of mycelium growth and sporangia production on the needle surface. From the detached-needle assay, we obtained three measured variables (observables): isolation rate (*R*_*isol*_, Eq 5), which is obtained by isolation of pathogen from needle fragments into culture media; presence of mycelium on the needle surface (*R*_*myc*_, Eq 6) and presence of sporangia (*R*_*spor*_, Eq 7), observed using fluorescence microscope. Those observables relate to the live pathogen on the needle, *Q*.

$$R_{isol}=c_{i}Q$$ (5)

$$R_{myc}=c_{m}Q(t-\tau_{m})$$ (6)

$$R_{spor}=c_{s}Q(t-\tau_{s})$$ (7)

where *c*_*i*,*m*,*s*_ are the detection responses for the observables, i.e. the experimental test sensitivities, and *τ*_*m*_ and *τ*_*s*_ are the time delays between infection and the growth of mycelium and sporangia(i.e. incubation time), respectively. In the detached-needle assay, we assumed that the time delay between growth of mycelium on the needle surface and sporangia production can be neglected as their emergence could not be differentiated in this instance. We assumed that this time delay was shorter than the time between our individual observations (sampling period of two days). Therefore, we assumed that *τ*_*m*_ = *τ*_*s*_ in the detached-needle assay. The incubation periods have typically taken the form of Weibull, Frechet or gamma distributions, i.e. long-tailed distributions with an asymmetric peak and support on the positive domain [35]. We modelled the incubation time probability density *p*(*τ*_*m*_) by a Frechet distribution with a shape parameter of 2 (Eq 8) because it yields approximately the right shape (see Fig 3), and the scale and location parameters provide enough degrees of freedom to fit the experimental observations.

$$p(\tau_{m})=\frac{2}{\sigma }{\left(\frac{t-\mu }{\sigma }\right)}^{-3}{exp}^{-{\left(\frac{t-\mu }{\sigma }\right)}^{-2}}$$ (8)

We report the mode of this distribution as the fit parameter *τ*_*m*_.

The empirical conditions of the detached-needle assay imposed two additional assumptions. As the inoculation took place in a fixed number of needles, the needle birth rate was assumed to be zero (*b* = 0). Furthermore, the dip inoculation procedure implied that needles were saturated with spores, i.e. at the maximum carrying capacity of the needles. Hence, we used *P* = *P*_*max*_ along the whole experimental period of 11 days. From that assumption, we resolved that infection dynamics was only due to primary infection. Finally, we also assumed that all the infected needles can produce isolates.

#### Fitting the detached-needle assay experimental data

The initial conditions to solve the model were *S*_0_ = 1 and *Q*_0_ = 0. The ordinary differential equations for *S* and *Q* (Eqs 1 and 3) can be solved analytically (see Supplementary material).

We used a Bayesian approach to estimate parameter values and their uncertainties [36]. The likelihood function given our model $\hat{y}$ with modelled Gaussian errors with standard deviation *σ* added is given in Eq 9.

$$L(\{t_{i}\},\{y_{i}\}|k,n,c_{m},\tau_{m})\propto (2\pi \sigma^{2}{)}^{-\frac{N}{2}}\prod_{i=1}^{N}exp\left[\frac{-(y_{i}-\hat{y}(t_{i}|k,n,c_{m},\tau_{m}){)}^{2}}{2\sigma^{2}}\right],$$ (9)

where *N* is the number of data points, and *y*_*i*_ represents and observable. The parameters *k*, *n* and *τ*_*m*_ need to be positive and, to make the fit process converge, it is helpful that they are bounded. The parameter *c*_*m*_ is a detection response and must range 0–1 by definition. Hence, based on our biological understanding from previous work and inspection of our raw experimental data, we used bounds that are at least 4 times larger than values we can realistically expect, the prior distribution contains the following bounds on parameter values: *k* and *n* in (0, 0.3), *c*_*m*_ in (0, 1), *τ*_*m*_ in (0, 6) and *σ*>0. Within those bounds, we used a flat prior. We used an Affine Invariant Markov chain Monte Carlo (MCMC) sampler [37], provided in the emcee package [38], to sample the posterior distribution. We used 100 sampling chains, initialized by adding 20% random noise around the maximum likelihood estimate of parameter values, with 15000 steps of which the first 1000 were burn-in samples. At each step, we solved $\hat{y}$ by numerical integration over *p*(*τ*_*m*_).

To verify that MCMC sample was well-behaved, we calculated the parameter autocorrelation lengths. The autocorrelation lengths were 760 samples, i.e. chain length was 18 independent samples, for parameter *k*; and in the range of 50–230 samples, i.e. > 60 independent samples, for all the other parameters. Jump acceptance fractions were in the range of 0.28–0.32 for all the chains. For more details and plots of all parameter covariances, see the Supplementary material.

#### Applying the model to the in vivo inoculation

The experimental observables for the *in vivo* inoculation are the isolation rate, which in this experiment is the proportion of pathogen detected by isolation (*R*_*isol*_, Eq 10) and by qPCR (*R*_*qpcr*_, Eq 11), as well as the pathogen-to-host DNA ratio from qPCR and sporangia (*R*_*dna*_, Eq 12).

$$R_{isol}=c_{i}Q$$ (10)

$$R_{qpcr}=c_{q}Q$$ (11)

$$R_{dna}=c_{d}Q$$ (12)

The assumptions made for the detached-needle assay are valid for the *in vivo* inoculation, with one exception. The spore density is not constant in the *in vivo* inoculation, hence *P* is variable (Fig 1C, Fig 3D). In this experiment, we thoroughly sprayed the plants with a suspension of zoospores. The amount of spores reaching the needles that were subsequently sampled is not defined. It cannot be assumed that spore density was at the maximum carrying capacity of the needles at the time of inoculation. Further, across the assay, spore density changes due to secondary infection, which in turn may decline with time [17].

In the *in vivo* inoculation, we cannot model *τ*_*m*_ because we did not perform microscopic observations of mycelium and sporangia (*R*_*myc*_,*R*_*spor*_). Instead, we used the parameter (*g*) in Eq 2 to determine the spore birth rate. Like in the detached-needle assay, we assumed that the needle birth rate (*b*) was zero for the 28-day period the experiment spanned. Indeed, we did not observe development of new shoots and needles, as the fog-room conditions of temperature, photoperiod and humidity were chosen such that they resemble as closely as possible a New Zealand winter season.

#### Fitting the in vivo inoculation experimental data

The initial conditions to solve the model were *S*_0_ = 1 and *Q*_0_ = 0. The state variable *P*_*o*_ was unknown, as the inoculum was sprayed on the plant. Therefore, *P*_*o*_ was a fitted parameter. We observed that our measurements exhibit heterocedasticity. We modelled this with additive and multiplicative Gaussian terms, to account for both constant errors and contributions that are proportional to the pathogen concentration (Eq 13).

$$\Delta y_{i}=\left(0.4+\frac{y_{i}}{max(y)}\right)\sigma$$ (13)

The number of time series data points and the amount of noise did not allow the determination of *m*. Simulations showed that a minimum of 20 data points would be required to determine *m* (see Supplementary material). Thus, we fixed *m* to a constant value of 0.15, which is biologically reasonable and lays in the middle of the range of values for *m*, for which good model fits can be obtained.

Taking into account all previous considerations, the model followed Eqs 1, 3, 4, 14 (replacing Eq 2) and 15 (replacing Eq 9).

$$\frac{dP}{dt}=gI\left(1-\frac{min(P,P_{max})}{P_{max}}\right)-0.15P$$ (14)

$$L(\{t_{i}\},\{y_{i}\}|k,n,g,P_{0},c_{i,q,d},\sigma )\propto (2\pi \sigma^{2}{)}^{-\frac{N}{2}}\prod_{i=1}^{N}exp\left[\frac{-(y_{i}-\hat{y}(t_{i}|k,n,g,P_{0},c_{i,q,d},\sigma ){)}^{2}}{2{\left(\Delta y_{i}\right)}^{2}}\right],$$ (15)

Similarly to the detached needle assay, we used a flat prior, with the following bounds: *k* in (0, 0.05), *n* in (0, 1), *g* in (0, 50), *P*_0_ in (0, *P*_*max*_), *c*_*i*,*q*,*d*_ in (0, 1), and *σ*>0. The MCMC sampler did not converge well due to the limited amount of data. The parameter autocorrelation lengths were on the order of the chain length. However, jump acceptance fractions were acceptable, in the range of 0.25–0.42 for all the chains. We verified that the parameter estimates agreed with the maximum likelihood estimate and with brute force optimization. Thus, estimates and confidence intervals should be reliable. For more details, see Supplementary material.

## Results

### Detached-needle assay: Comparing susceptible and resistant genotypes

**Fig 4:**
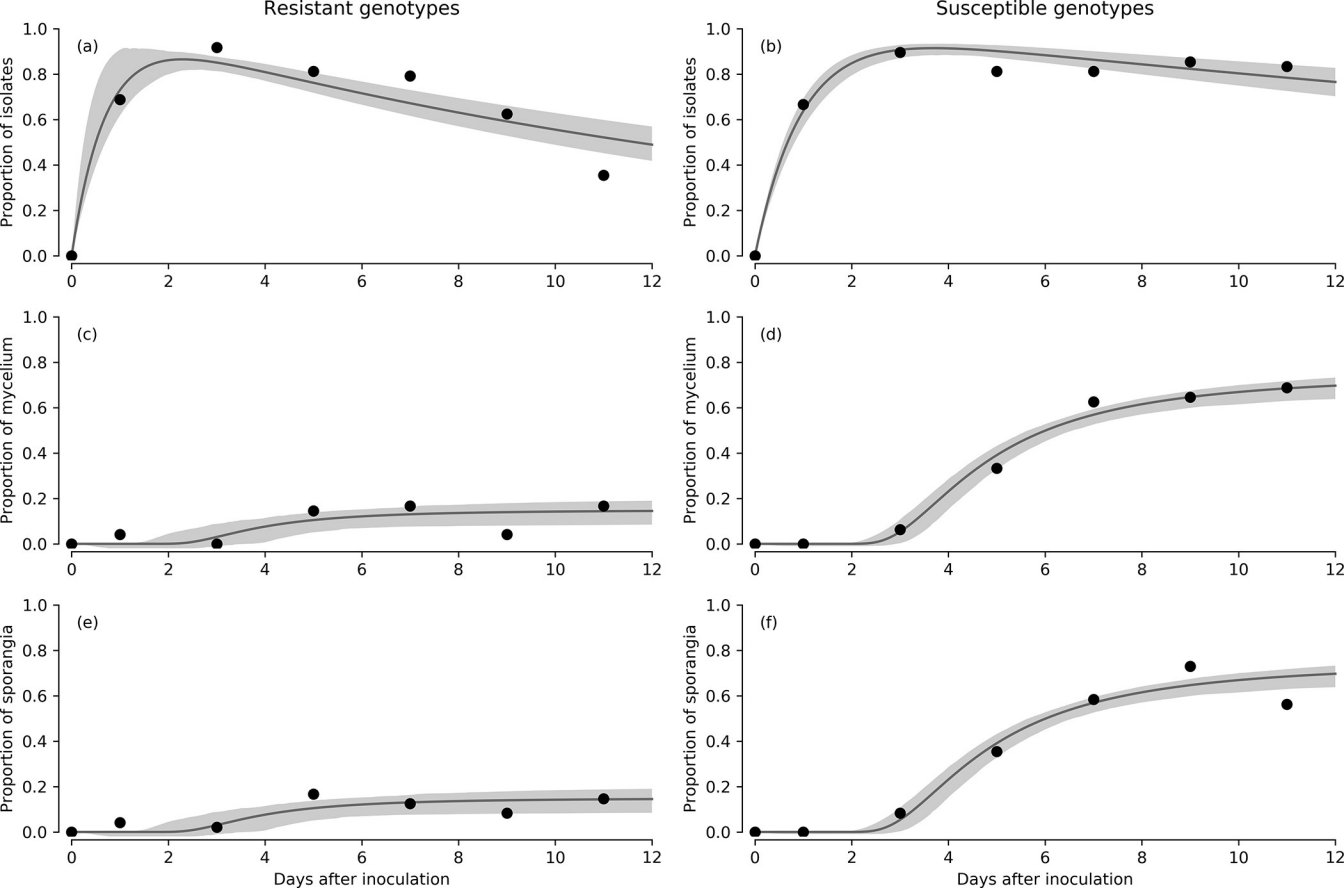
Time series data for the isolates (a-b), mycelium (c-d) and sporangia (e-f) proportions from the detached-needle assay for resistant (a, c, e) and susceptible (b, d, f) genotypes, with the SIPQ model fits to the data. Shaded areas are the 95% credible regions of the fit.

The model fit for the detached-needle data is shown in Fig 4. The infection rates and the incubation times were similar for both resistant and susceptible genotypes (Table 3). Pathogen death rate was 2.5 times higher for resistant than susceptible genotypes (Fig 4A and 4B, Table 3). Further, only 28% of the resistant ramets produced mycelium and sporangia, whereas 90% of the susceptible ramets did (Table 3).

**Table 3:** Model parameter estimates (median) for the detached-needle assay with 95% credible regions.

Parameter	Resistant genotypes	Susceptible genotypes
*k*	$0.014_{-0.004}^{+0.017}$	$0.010_{-0.002}^{+0.002}$
*n*	$0.063_{-0.012}^{+0.014}$	$0.024_{-0.007}^{+0.008}$
*c*_*m*_	$0.28_{-0.12}^{+0.35}$	$0.90_{-0.17}^{+0.09}$
*τ*_*m*_	$3.2_{-1.6}^{+1.5}$	$3.7_{-0.4}^{+0.4}$

**Fig 5:**
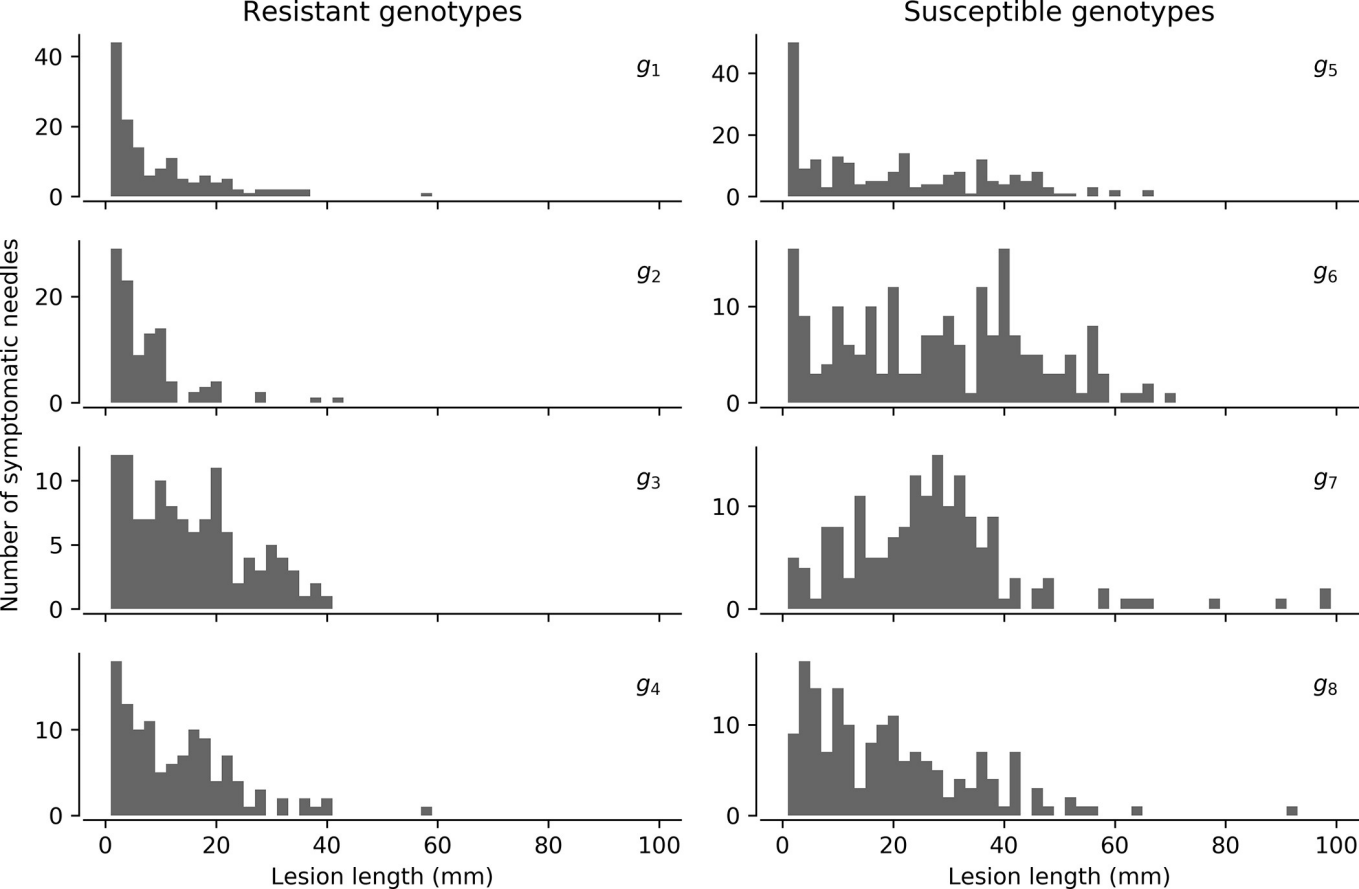
Histograms of lesion lengths per genotype for four resistant (a, c, e, g) and four susceptible (b, d, f, h) genotypes in the detached-needle assay.

**Fig 6:**
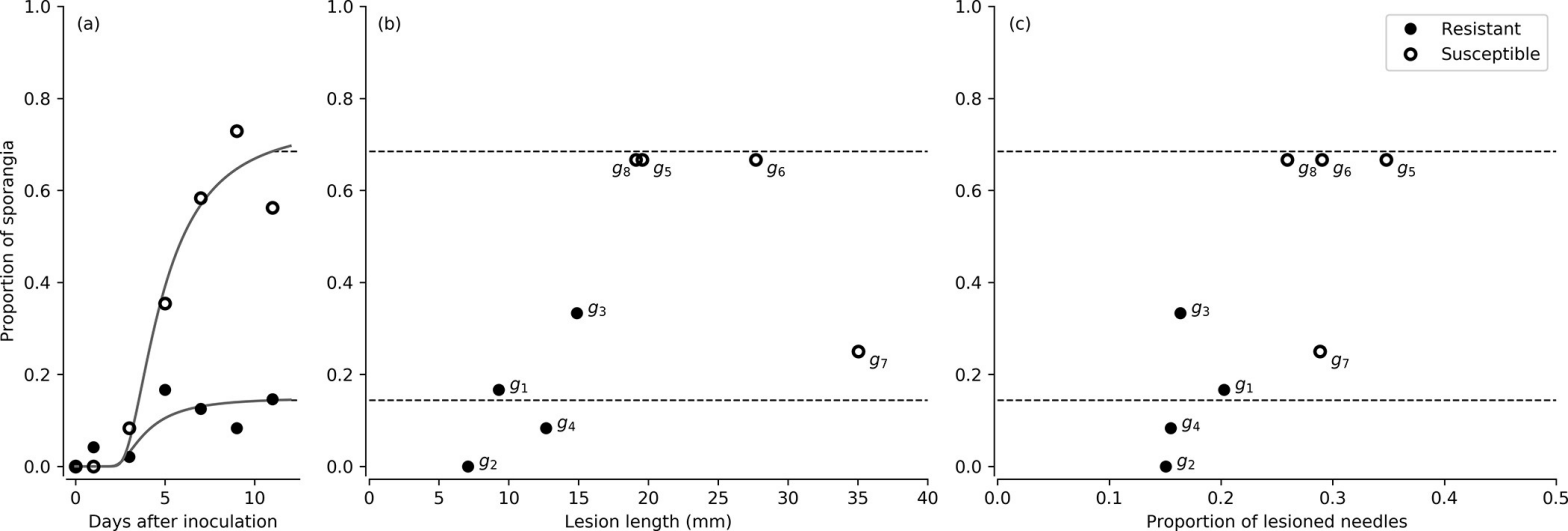
Connecting the model fit for sporangia proportion (a) to the lesion lengths (b) and the proportion of needles with lesions (c), both at the last time point in the time series (11 days). This is the only point at which the lesions were measured.

The distribution of lesions lengths also differed between resistant and susceptible genotypes (Fig 5). In resistant genotypes, small lesions and no lesions appeared to be most frequent, while in susceptible genotypes, lesion lengths spread from 0 to 100 mm (Fig 5). Susceptible genotypes presented higher sporangia production (Fig 6A), which grouped with higher lesion lengths, except for g7 which showed low sporangia production despite the highest lesion length magnitude within susceptible genotypes (Fig 6B). Sporangia production was also related to proportion of needles with lesions, showing susceptible and resistant genotypes grouped apart, except for susceptible g7 (Fig 6C).

### *In vivo* inoculation

**Fig 7:**
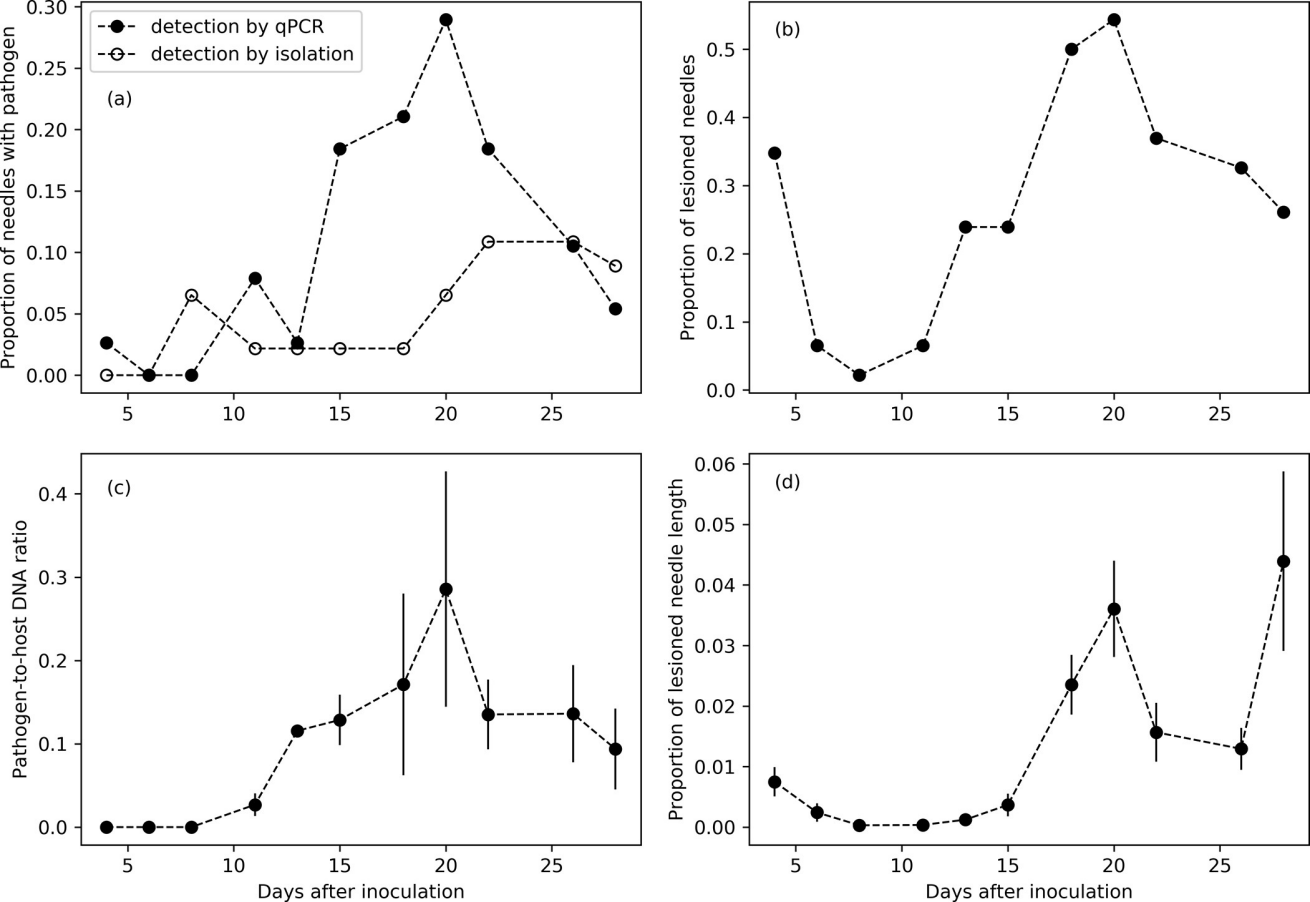
Time series data for the proportion of needles with live pathogen (a) detected by qPCR and isolations, proportion of needles with lesions (b), pathogen-to-host DNA ratios (c) and proportion of lesioned needle length (d) averaged per time point in the *in vivo* inoculation. Symbol and bars represent means ± standard error.

**Fig 8:**
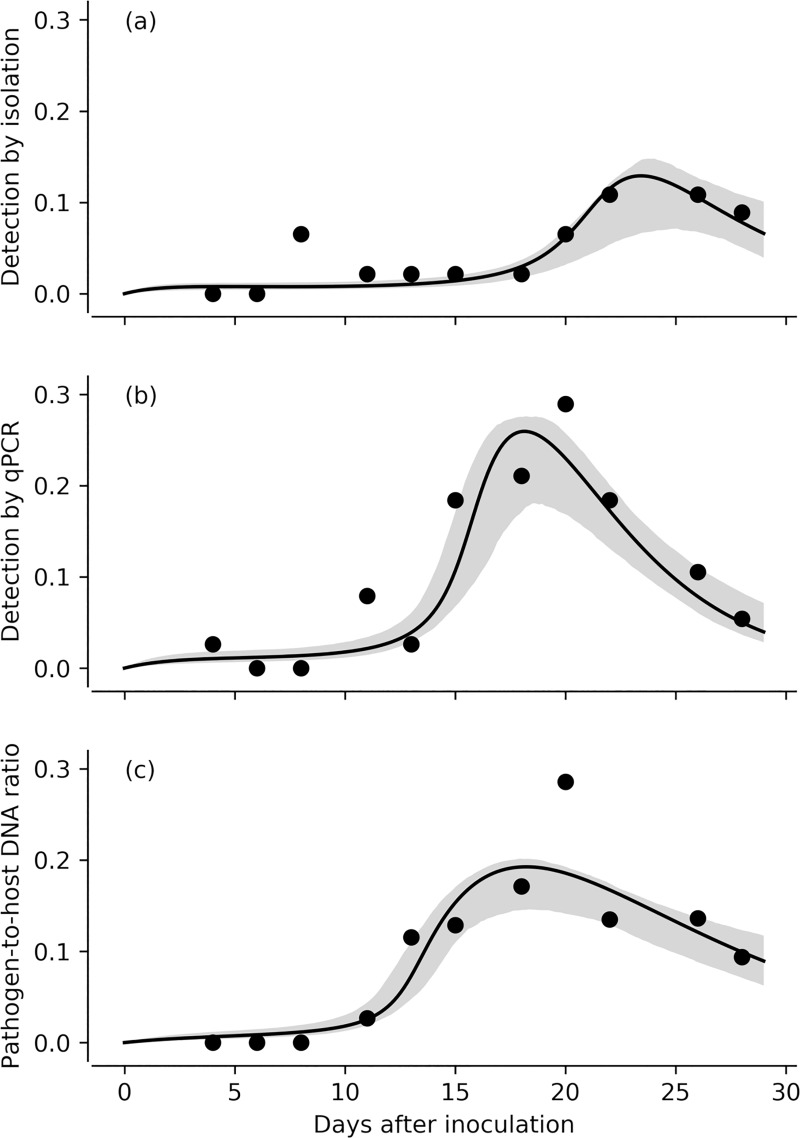
SIPQ model fits for the proportion of needles with live pathogen detected by isolations (a), qPCR (b), and the pathogen-to-host ratio (c) for the *in vivo* inoculation. Shaded areas are the 95% credible regions of the fit.

The proportion of needles with live pathogen (detected by isolations and qPCR) and with lesions and the proportion of lesioned needle length presented a first peak at four days after inoculation (Fig 7A, 7B and 7D). Detection by qPCR and pathogen-to-host DNA ratio were maximum at 20 days after inoculation, and then decreased (Fig 7A). In contrast, the peak of detection was reached at 22 and 26 days after inoculation by isolation (Fig 7A). Detection by qPCR was more effective and showed an earlier and higher peak than isolations (Figs 7A, 8A and 8B). Pathogen-to-host DNA ratio peaked at a similar time to the proportion of needles with live pathogen (Figs 7A, 7C, 8B and 8C). The estimation of *n* is mainly determined by the negative slope of *Q* after the maximum live pathogen proportion has been reached (as illustrated in Fig 8C). As data of detection by isolation has only two time points after that peak, the fit for detection by isolation is the least well behaved, in particular for *n* (see Supplementary material).

The infection rate (*k*), the spore birth rate (*g*) and the proportion of needles with live pathogen (detection response, *c*_*i*,*q*,*d*_) were predicted to be higher when qPCR methods were used to assess infection, compared to isolation (Table 4). Further, the pathogen death rate (*n*) and the carrying capacity of spores at inoculation were predicted to be higher when isolation was used to assess infection. However, the 95% credible regions were wide with overlap between the three methods compared. This is due to the limited number of data points in the time series data, and the relatively large amount of noise per point (see standard errors bars in Fig 7C and 7D).

**Table 4:** Model parameter estimates (median) for the *in vivo* inoculation with 95% credible regions for infection rate (*k*), pathogen death rate (*n*), spore birth rate (*g*), carrying capacity of spores for each needle at inoculation time (*P*_*0*_) and the detection response for the observables (*c*_*i*,*q*,*d*_).

Parameter	Isolation	qPCR	DNA ratio
*k*	$0.0041_{-0.0029}^{+0.0086}$	$0.0056_{-0.003}^{+0.018}$	$0.0036_{-0.0022}^{+0.016}$
*n*	$0.54_{-0.47}^{+0.41}$	$0.36_{-0.23}^{+0.38}$	$0.16_{-0.13}^{+0.48}$
g	$4.5_{-2.8}^{+7.3}$	$7.4_{-5.1}^{+10}$	$12_{-9.4}^{+20}$
*P*_0_	$1.9_{-1.2}^{+3}$	$1.2_{-0.81}^{+1.7}$	$1.4_{-0.94}^{+2.1}$
*c*_*i*,*q*,*d*_	$0.82_{-0.64}^{+0.88}$	$0.94_{-0.6}^{+0.92}$	$0.53_{-0.34}^{+1.2}$

#### Assessment of quantification methods and symptom expression

**Fig 9:**
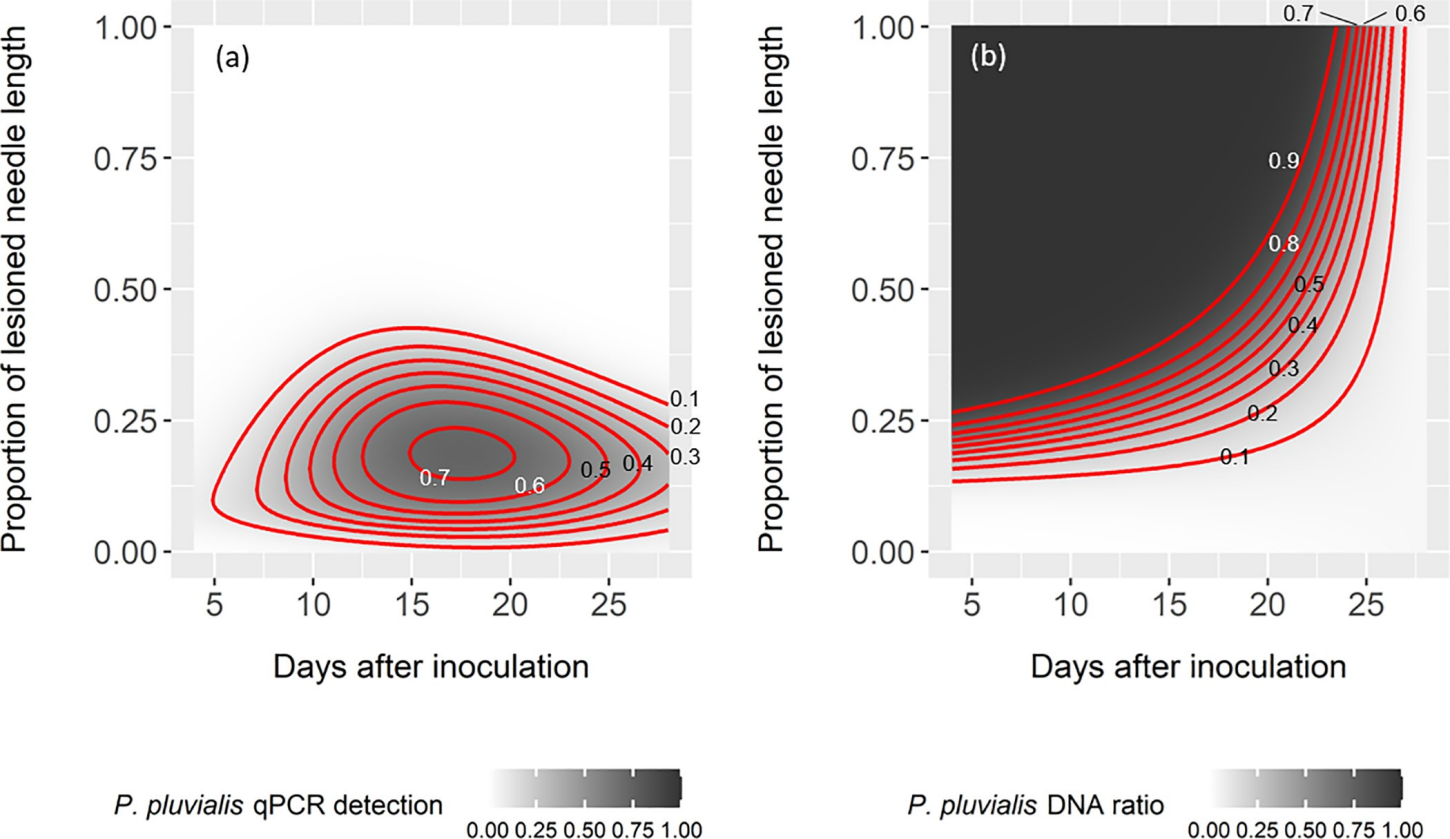
Predicted proportion of needles with live pathogen, i.e. *P*. *pluvialis* qPCR detection rate (a), and *P*. *pluvialis* DNA ratio (b) as a function of days after inoculation and the proportion of lesioned needle length. Predictions were derived from generalized additive mixed effects models applied to the *in vivo* inoculation data. Both axes are statistically significant at P = 0.05.

For the *in vivo* inoculation, we complemented the model fits with GAMM models to analyse the link between quantification methods (isolation and qPCR) and lesion length. The GAMM models showed an increase of the isolation rate across time and no effect of lesion length on isolation success (Table 5). In contrast, detection of *P*. *pluvialis* through qPCR did correlate with the proportion of lesioned needle length, with a maximum of qPCR detection rate of 19% symptomatic tissue (total needle length) at 18 days after inoculation, according to the predictions of the GAMM model (Table 5, Fig 9A). *P*. *pluvialis* abundance on the needles measured as pathogen-to-host DNA ratio decreased with both days after inoculation and the proportion of lesioned needle length (Table 5, Fig 9B).

**Table 5:** Results from the best-fit generalized additive mixed models for the isolation rate, qPCR detection rate, and *P*. *pluvialis* abundance measured as pathogen-to-host DNA ratio. Dai: Days after inoculation; lesion needle: proportion of lesioned needle length; edf: estimated degrees of freedom; t2: bivariate smooth term.

	Isolation rate Family: binomial Link: log			qPCR detection rate Family: binomial. Link: logit			*P*. *pluvialis* abundance Family: tweedie (1.44). Link: log		
**Parametric coefficients**	**Estimate (SE)**	**z**	**P**	**Estimate (SE)**	**z**	**P**	**Estimate (SE)**	**z**	**P**
Intercept	-4.96 (0.71)	-6.98	<0.001	-3.04 (0.31)	-9.78	<0.001	-4.83 (0.26)	-18.71	<0.001
Dai	0.10 (0.03)	3.02	0.003						
**Bivariate smooth terms**	**edf**	**Chi.sq**	**P**	**edf**	**Chi.sq**	**P**	**edf**	**F**	**P**
t2(Dai, lesion needle)				6.79	23.73	0.003	3	23.88	<0.001

## Discussion

In this study, we developed an extended RNC epidemiological model, which was tested against experimental data for susceptible and resistant genotypes to parameterize the infection drivers in both detached-needle and *in vivo* inoculations. Moreover, we scaled up our epidemiological model to analyze the infection dynamics for susceptible genotypes at the whole-plant level. With this *in vivo* inoculation data, we also contrasted the methods of detection used in assessing infection in relation to symptom expression. The extended model introduced the state variable *Q* which allowed us to account for the dynamics of the pathogen on the needle. That is particularly important for the polycyclic RNC disease. This model fits presented here provided insights into the drivers of the RNC epidemic. Whole-plant infection dynamics identified the challenge of high noise in the sampling points, but indicated that the detection method applied to assess disease was the single most important factor influencing parameter estimates.

### Comparing susceptible and resistant genotypes

The detached-needle assay was carried out under laboratory conditions, which favoured infection and pathogen development through maximum pathogen load and minimizing defence ability to fight disease by needles detached from stems. Thus, the susceptible plant material in this experiment provided optimal conditions for the pathogen and the maximum likely values for *k* and minimum values of *τ*_*m*_.

While infection rates (*k*) and incubation times (*τ*_*m*_) were similar between susceptible and resistant genotypes, the pathogen death rate (*n*) was 2.5 times higher for resistant genotypes (Table 2). Mycelium and sporangia were produced extensively in susceptible, but not in resistant genotypes (Table 2). Therefore, in resistant genotypes, the pathogen was observed to infect, but seldom produced reproductive structures. Fewer and smaller lesions were produced by the pathogen in the resistant genotypes (Figs 5 and 6), suggesting that the survival of the pathogen is limited within the necrotrophic phase in the resistant genotypes. Lesion development appeared to align with sporulation, with low levels of both in resistant genotypes (Fig 6). This indicates that the necrotrophic phase of *P*. *pluvialis* may be linked to the sporulation of the pathogen. The incubation time was surprisingly not linked to disease resistance, and was either constant across host individuals, or dependent on other unknown factors. The differences detected through the SIPQ model between resistant and susceptible genotypes suggested a stronger defence strategy or pathway in resistant genotypes, which may be informative for disease resistance screening and applied management of the disease. A better understanding of the defence response in resistant genotypes should be given attention in further research.

The detached-needle assay model fit reflected the primary infection rate of the pathogen, as the experimental design did not allow for secondary inoculum production and infection of newly emergent needles. The peak of the infection took place 3 days after inoculation (Fig 4A and 4B) and was similar in both susceptible and resistant genotypes. This peak was in conditions optimal for pathogen development, and may well occur later in the field.

### Scaling up to whole-plant infection dynamics

In our *in vivo* inoculation, we aimed to describe the sustained infection dynamics of whole plants to account for variable amounts of inoculum across primary and secondary infection and their respective transmission rates being closer to what occurs naturally in the field. We commenced disease monitoring 4 days after inoculation. Pathogen isolation, qPCR detection rate and lesion development each presented dual peaks. For qPCR detection and lesions, the first peak occurred at the first sampling point (4 dai), while for isolations the primary peak was observed at the second sampling point (6 dai) (Fig 7A, 7B and 7D). A distinguishable peak for primary infection has been previously reported in other epidemiological studies [17,18]. Having distinguished this primary infection cycle, we were able to quantify the polycyclic infection by *P*. *pluvialis*. Based on the *in vivo* experiment, re-infection cycles will occur within 4–6 days. The peak at 6 days after inoculation detected by isolations in the *in vivo* inoculation was delayed by three days compared to the detached-needle assay (same detection method). This lag in the *in vivo* inoculation trial is most likely attributable to more vigorous defences afforded by the intact physiological connection between needles and the remaining plant body in contrast to detached needles.

Following secondary infection and the resultant second peak of the epidemic, the transmission rate detected by qPCR decreased more quickly compared to isolations. Secondary infection has been recognized to slow down due to several factors. First, there is a decrease in availability of uninfected susceptible plant material [17] so that the probability of the spores reaching healthy needles decreases. This is particularly true for this study in which the experimental conditions simulated the winter season. In these conditions, over the course of the experiment, no new flush was observed and hence no new needles were available for infection. Secondly, the susceptibility of plants decreases due to the systemic acquired resistance. This process confers a stronger defence response upon secondary infection to leaves distal to the primary focus of infection [39,40]. Finally, needle age may also have a role in modulating the transmission rate of infection. RNC disease has been recognised to preferentially infect fully mature needles [9]. In our experiment, we observed this preference, however, current-year needles also became infected under high infection pressure. We presume that current-year needles may be better defended than older needles, in agreement with the optimal defence theory [41], slowing down pathogen development. Our experimental design did not allow us to distinguish between primary and secondary infection at the needle scale. However, the pattern of infection is consistent with polycyclic infection observed for *Phytophthora* pathogens and *P*. *pluvialis* epidemiology observed to date. We can therefore infer that the first peak was due to primary infection, i.e. zoospore solution being the source of the inoculum, matching the detached-needle assay (approx. 3 days after inoculation), and the second peak was due to secondary infections from sporulating needles.

### Comparing *P*. *pluvialis* assessment procedures

Diagnostic methods seem to play a crucial role in defining the dynamics of *P*. *pluvialis* infection and associated development of RNC. In our method comparison (Table 4, Fig 8), the 95% credible regions overlap due to high noise in the sampling points. However, it is clear that the isolation and qPCR approaches lead to qualitatively different epidemic outputs and, moreover, the detection method also had the greatest influence on the parameter estimates in our models.

Regardless of the detection method, the infection level decreases after an early peak, whereas the proportion of lesioned needle length peaks at the end of the experiment. We observed that secondary infections due to other pathogens (*Dothistroma* sp., *Cyclaneusma* sp., *Pestalotiopsis* sp.) took place with lesions that occupied space on the needles. Even though the grafts were sanitized before the experiment, residual spores from other pathogens are likely to have remained on and within the well-established grafts and contributed to the proportion of necrotic tissue not available for infection by *P*. *pluvialis*. Detection by qPCR predicted an earlier second peak and a sharper decrease in the epidemic compared to isolations (Fig 8A and 8B). These differences can be explained by two factors. On the one hand, the latent detection by isolations may reflect a lack of intercellular invasion by *P*. *pluvialis* in the early stages of infection whereby surface sterilization may actually kill the mycelium established peripherally within the stomata. On the other hand, the low detection rates by qPCR at the later stages of the epidemic suggests that qPCR is more sensitive to the presence of secondary pathogens and/or dead cells than isolations. Indeed, proportion of lesioned needle length did not explain the variability in isolation rates, which was only explained by the days after inoculation (Table 4). In contrast, both *P*. *pluvialis* qPCR detection rate and DNA ratio were significantly influenced by both days after inoculation and lesion length (Table 4, Fig 9A). Detection by qPCR was optimal when 19% of the needle was lesioned with longer lesions associated with decreased qPCR detection (Fig 9A). While isolations measure the viability (outgrowth) of the pathogen, qPCR measures the presence of *P*. *pluvialis* DNA, being the pathogen either viable or dead. We argue that the timeframe of the experiment is short enough for the DNA to degrade. Hence, qPCR detection may include non-viable *P*. *pluvialis* inoculum. The proportions given by isolations and qPCR detection are proportions of infected needles, in contrast to *P*. *pluvialis* DNA ratio, which measured the average amount of DNA of *P*. *pluvialis* on the needles with respect to *P*. *radiata* DNA. This ratio was predicted to decrease with the days after inoculation, but was higher with larger lesion lengths (Fig 9B). Thus, in needles with lesions larger than the 25% of the needle length, detection rates are low (false negative), but, when positive, qPCR accurately detects high amounts of DNA, as expected (Fig 9A and 9B). This indicates a threshold-dependent accuracy for qPCR-based determination of *P*. *pluvialis* DNA.

The extension of the SI to the SIPQ epidemiological model provided substantial information about the drivers of RNC disease. In a polycyclic infection, it is important to include the proportion of infected needles with live pathogen on them, as well as the rates of pathogen birth and death. Thus, we were able to estimate the primary peak of the infection at the plant level to occur after 4 to 6 days (depending on the detection method), reflecting the period needed for *P*. *pluvialis* to establish re-infection. Our analyses showed that the choice of *Phytophthora* assessment method is key for epidemic prediction. qPCR was more sensitive than isolations earlier in the infection process, making it the best option for quantifying the early stages of disease epidemics. However, a combined detection method may yield the most realistic scenario. The SIPQ model showed promise for expanding its use to infer prediction models for the RNC disease. Silvicultural settings, edaphic and (seasonal) climatic conditions associated with *P*. *radiata* plantations can readily be incorporated into the SIPQ model allowing the identification and quantification of further disease-conducive factors.

## Supporting information

S1 Fig Experimental design for the detached-needle assay, only shown for control treatment. Each tray has five fascicles from one ramet from each genotype and is harvested at a certain date. There are six trays sampled at a certain date. The *P*. *pluvialis* inoculation treatment has the same implementation. The trays are randomly distributed in the space.
(TIF)

S2 Fig Experimental design for the *in vivo* inoculation experiment. All the plants are randomly distribute in each room. All the plants are sampled in each sampling date.
(TIF)

S3 Fig Posterior probability distributions of model parameters for the fit of the model with (*P = P_max_*) to the detached-needle assay data for resistant genotypes. (TIFF)

S4 Fig Fit of generated data with 30 time points. Solid black line is the model result for the parameter estimates. Thin lines are 400 randomly sampled lines from the full MCMC sampler chain.
(TIFF)

S5 Fig Posterior probability distributions of model parameters, for the fit of the model to the generated data with 30 time points. The histograms along the diagonal show the single parameter distributions, the off-diagonal plots show the covariances between parameters.
(TIFF)

S6 Fig Fit of generated data with the same number of time points as in the on-plant inoculation. Solid black line is the model result for the parameter estimates. Thin lines are 400 randomly sampled lines from the full MCMC sampler chain.
(TIFF)

S7 Fig Posterior probability distributions of model parameters, for the fit of the model to the generated data with the same number of time points as in the on-plant inoculation. (TIFF)

S8 Fig Posterior probability distributions of model parameters for the model fit to the *detection by isolation* data of the on-plant inoculation. (TIFF)

S9 Fig Posterior probability distributions of model parameters for the model fit to the *detection by qPCR* data of the on-plant inoculation. (TIFF)

S10 Fig Posterior probability distributions of model parameters for the model fit to the *pathogen-to-host DNA ratio* data of the on-plant inoculation. (TIFF)

S1 File (DOCX)
