# Supplementary figures and images for: Modelling the key drivers of an aerial Phytophthora foliar disease epidemic, from the needles to the whole plant
Source: PLoS One. 2019 May 28;14(5):e0216161. doi: 10.1371/journal.pone.0216161 (PMC6538149; doi:10.1371/journal.pone.0216161)

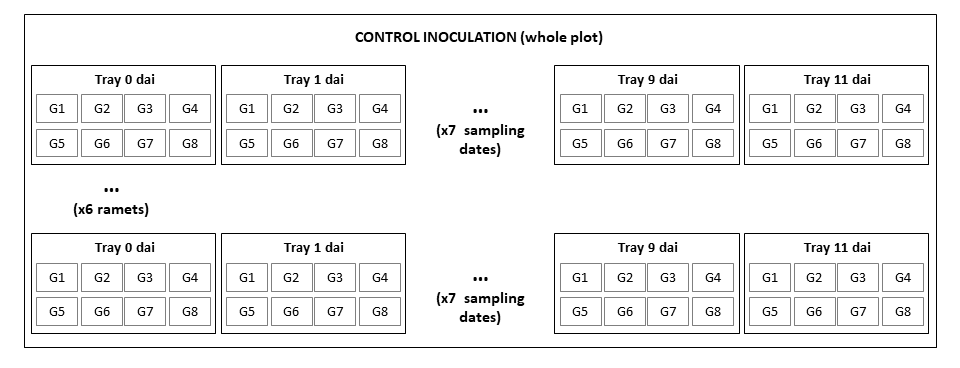

Supplement: S1 Fig — Each tray has five fascicles from one ramet from each genotype and is harvested at a certain date. There are six trays sampled at a certain date. The P. pluvialis inoculation treatment has the same implementation. The trays are randomly distributed in the space. (TIF) [file pone.0216161.s001.tif]

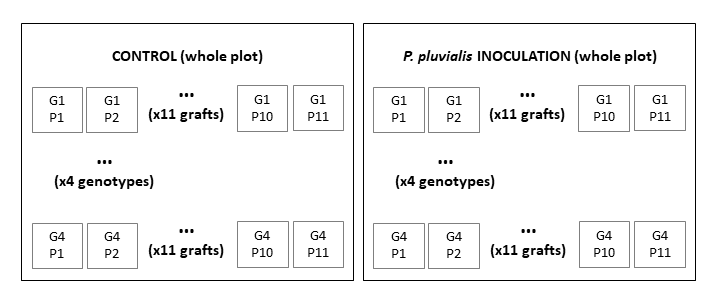

Supplement: S2 Fig — All the plants are randomly distribute in each room. All the plants are sampled in each sampling date. (TIF) [file pone.0216161.s002.tif]

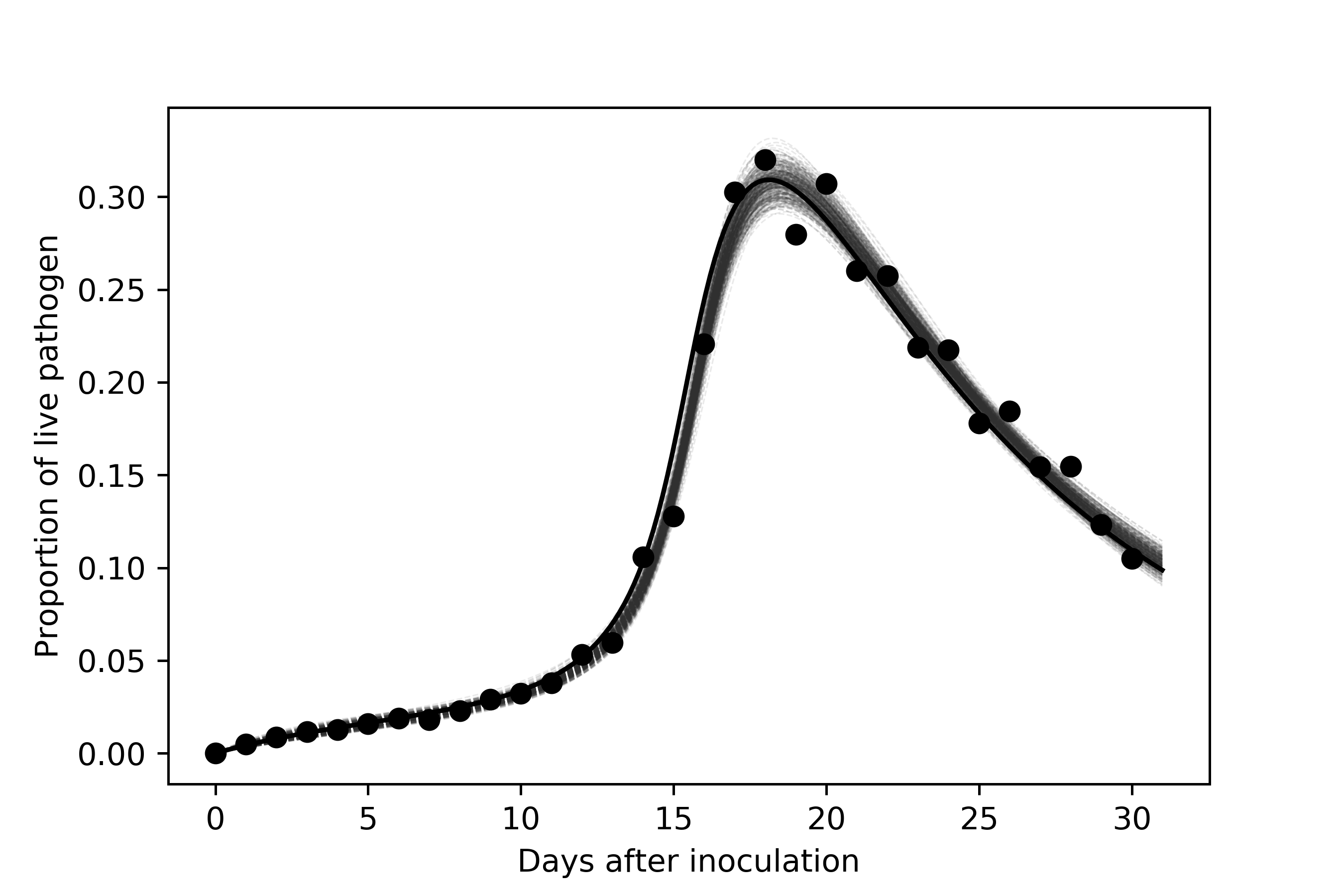

Supplement: S4 Fig — Solid black line is the model result for the parameter estimates. Thin lines are 400 randomly sampled lines from the full MCMC sampler chain. (TIFF) [file pone.0216161.s004.tiff]

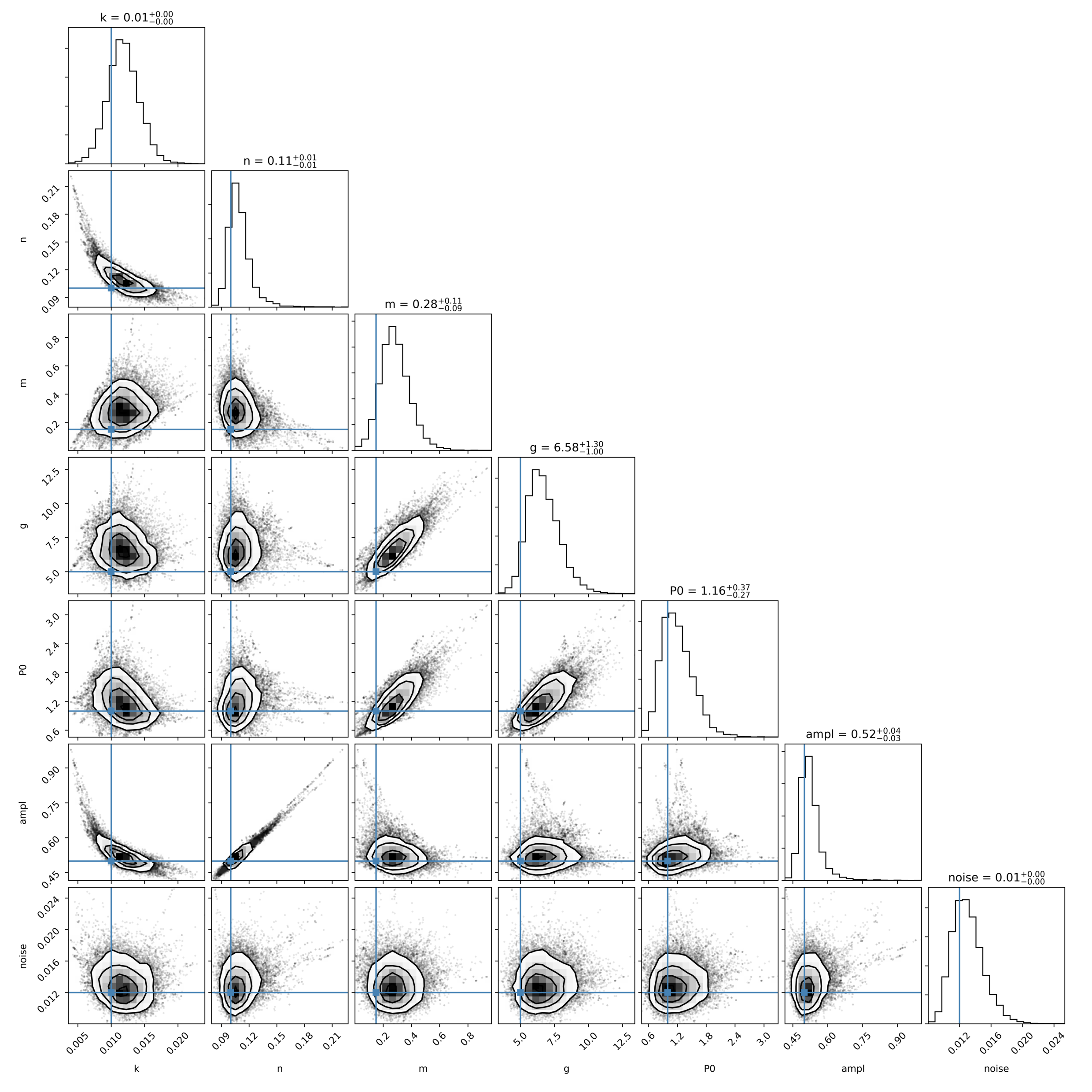

Supplement: S5 Fig — The histograms along the diagonal show the single parameter distributions, the off-diagonal plots show the covariances between parameters. (TIFF) [file pone.0216161.s005.tiff]

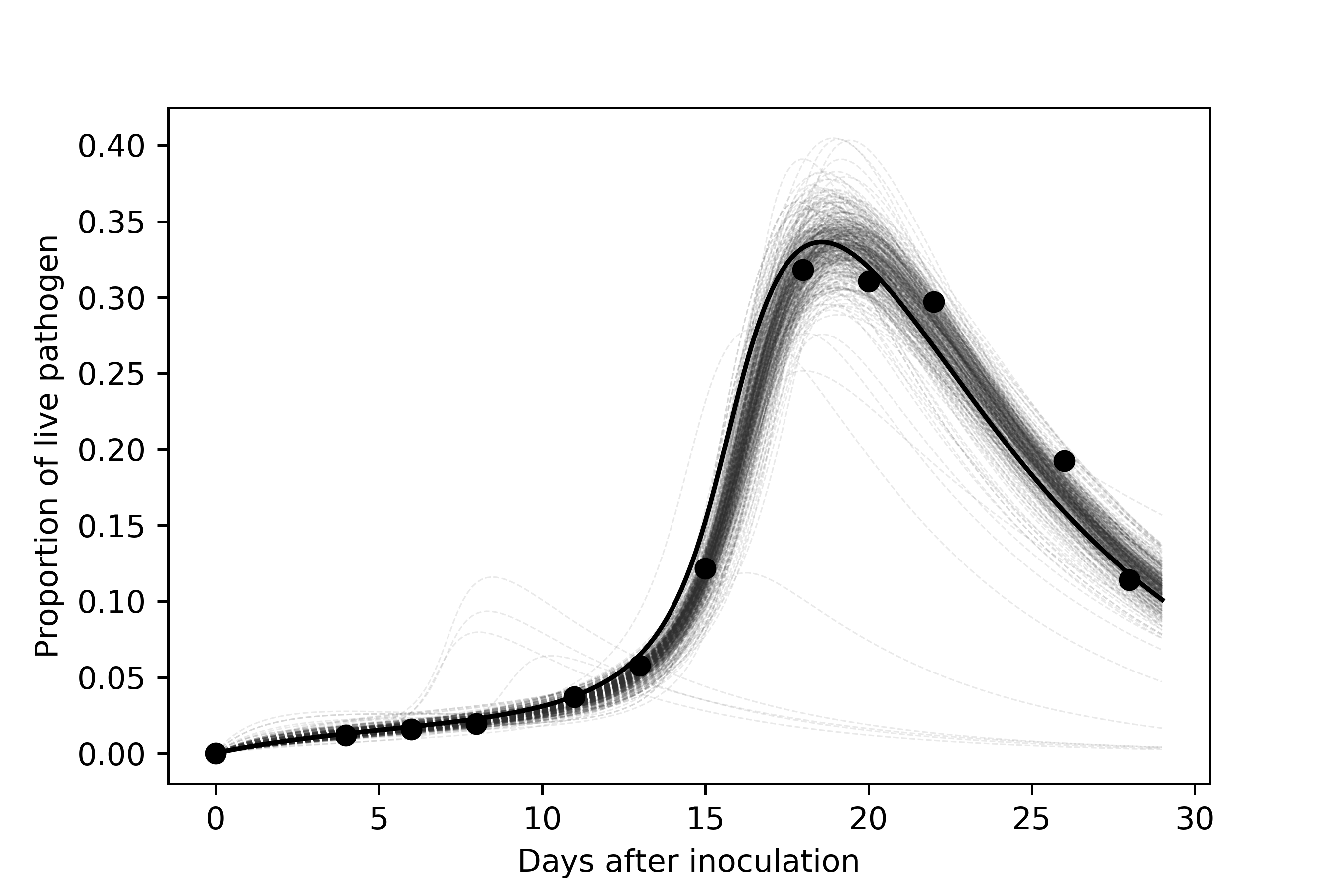

Supplement: S6 Fig — Solid black line is the model result for the parameter estimates. Thin lines are 400 randomly sampled lines from the full MCMC sampler chain. (TIFF) [file pone.0216161.s006.tiff]

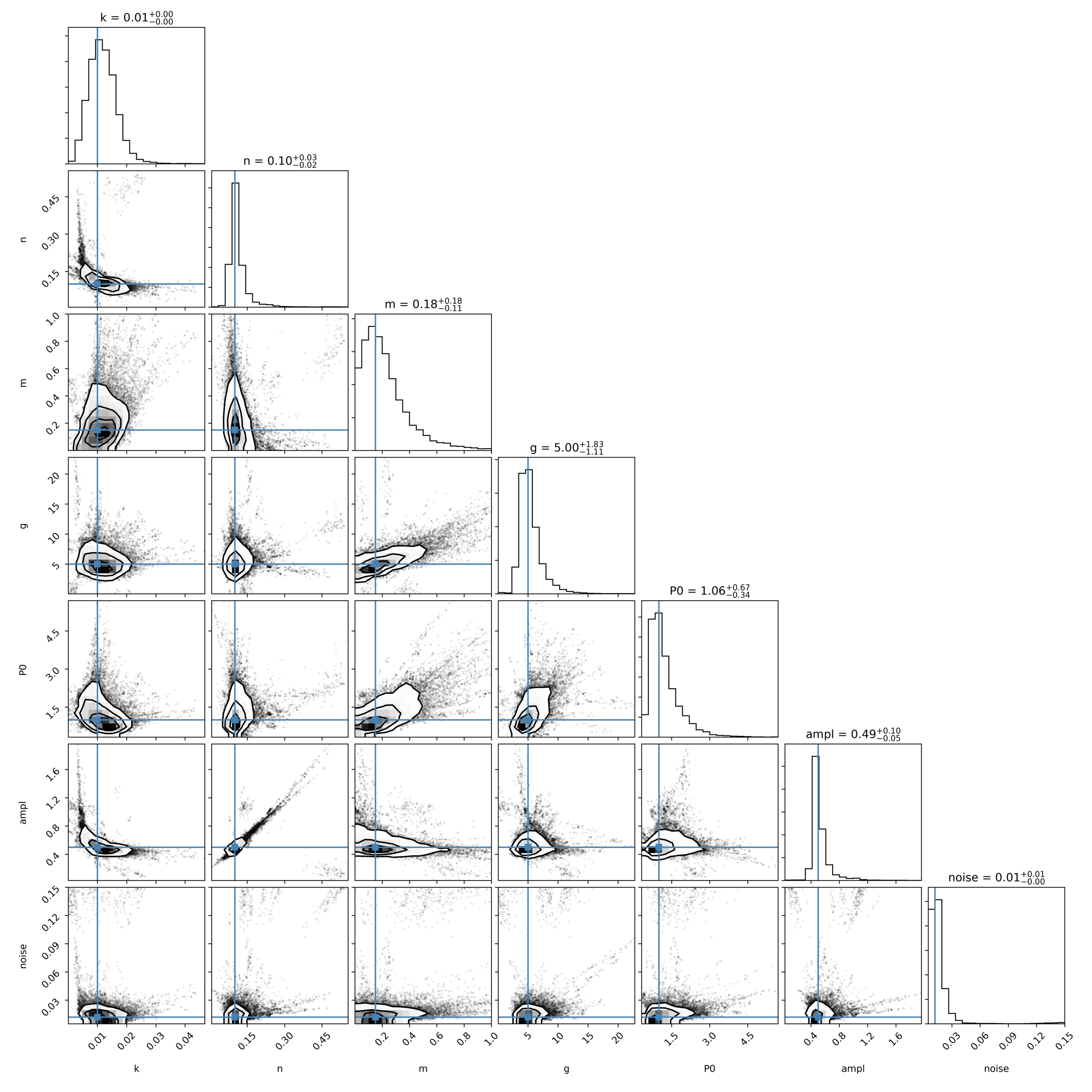

Supplement: S7 Fig — (TIFF) [file pone.0216161.s007.tiff]

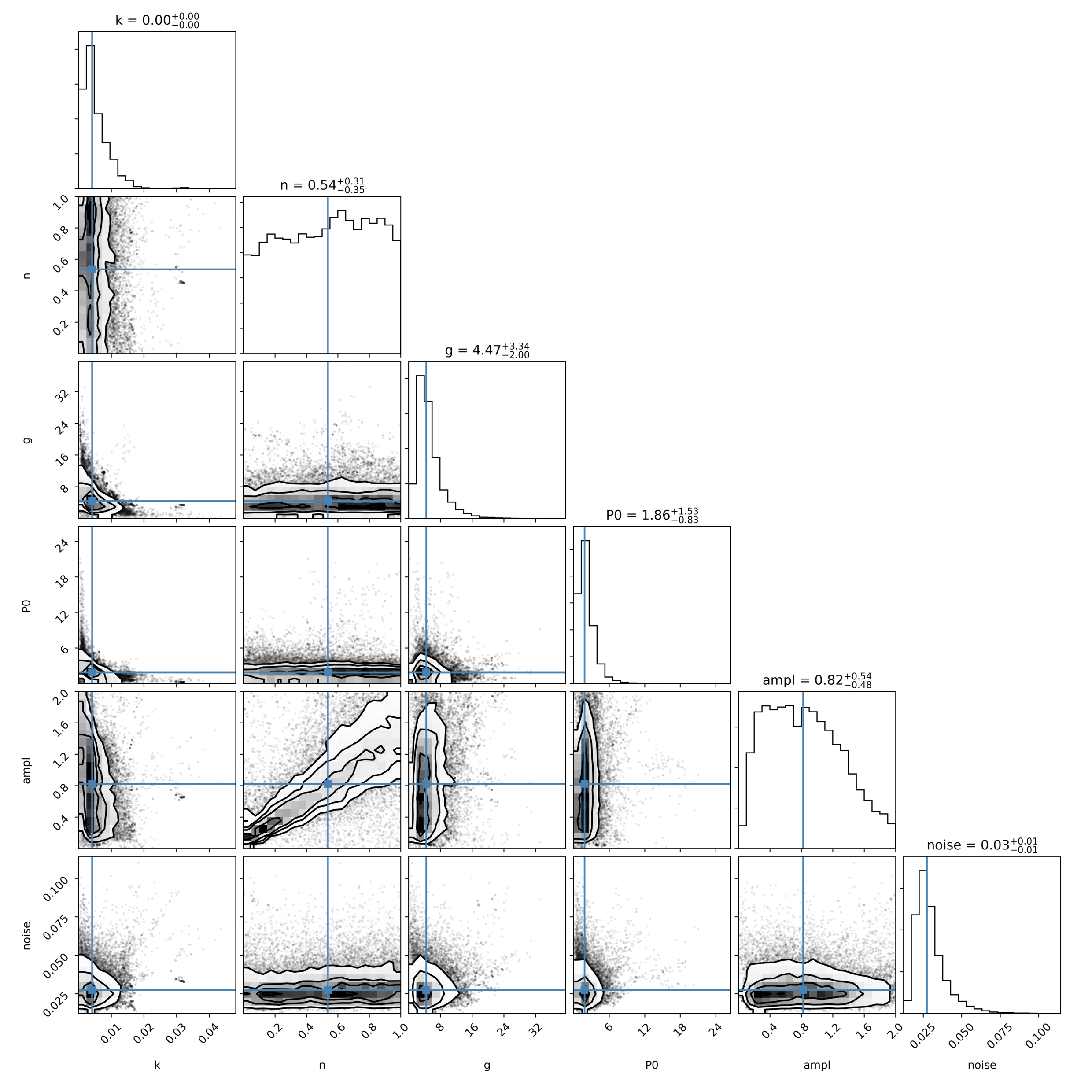

Supplement: S8 Fig — (TIFF) [file pone.0216161.s008.tiff]

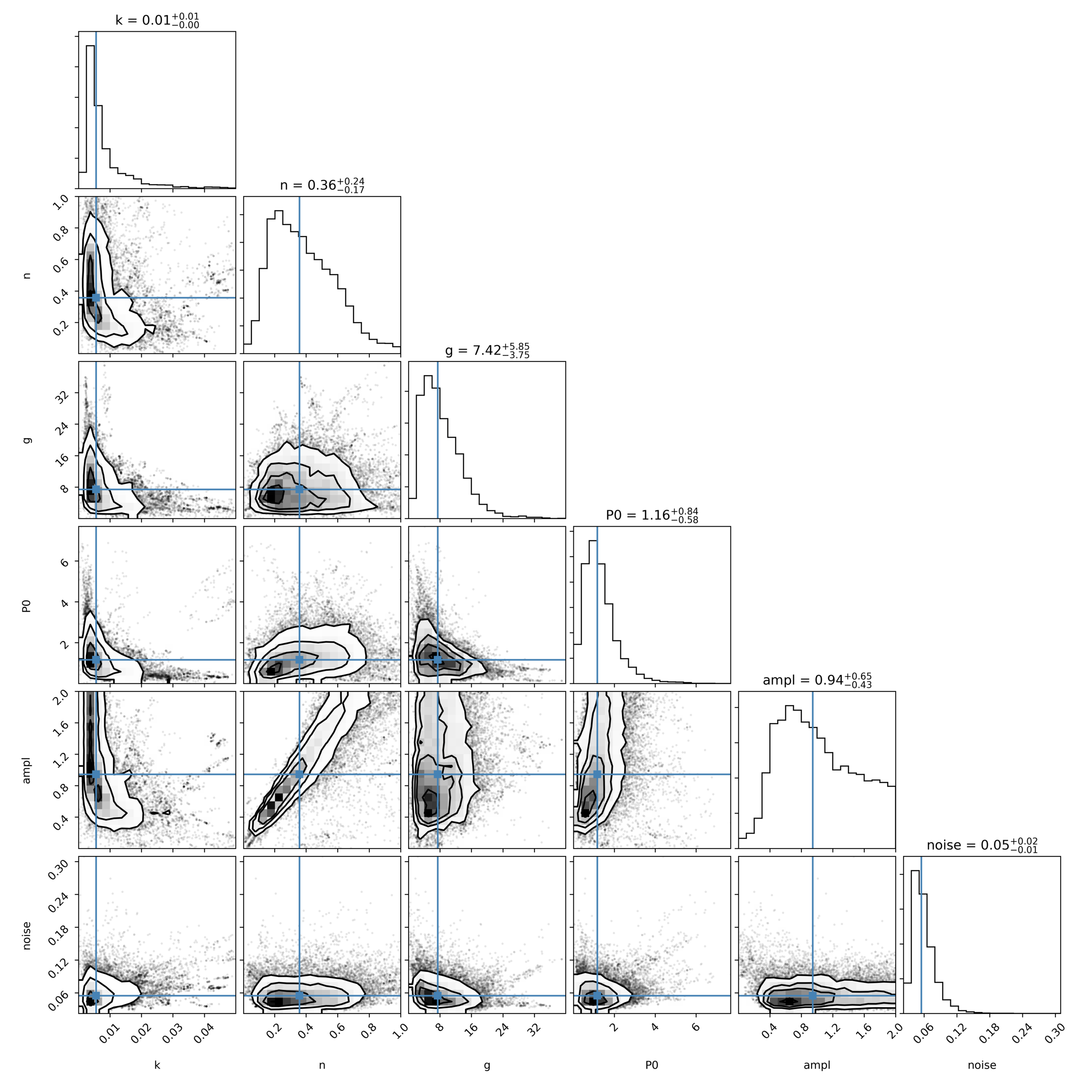

Supplement: S9 Fig — (TIFF) [file pone.0216161.s009.tiff]

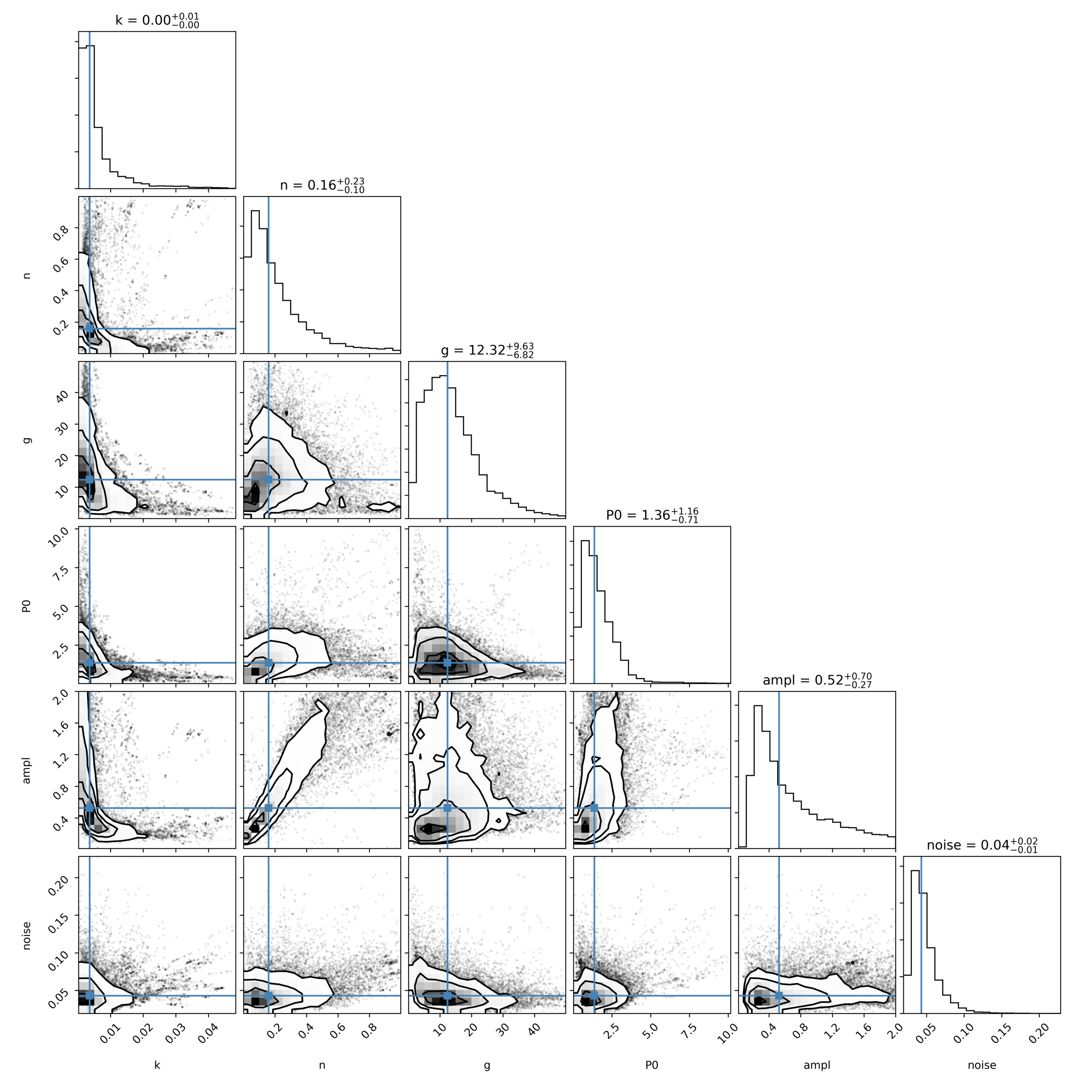

Supplement: S10 Fig — (TIFF) [file pone.0216161.s010.tiff]
