# Supplementary material for: Modelling the key drivers of an aerial Phytophthora foliar disease epidemic, from the needles to the whole plant
Source: PLoS One. 2019 May 28;14(5):e0216161. doi: 10.1371/journal.pone.0216161 (PMC6538149; doi:10.1371/journal.pone.0216161)
Supplement: S1 File — (DOCX) [file pone.0216161.s011.docx]

Supplementary material

Fig S1. ****Experimental design for the detached-needle assay, only shown for control treatment. Each tray has five fascicles from one ramet from each genotype and is harvested at a certain date. There are six trays sampled at a certain date. The *P. pluvialis* inoculation treatment has the same implementation. The trays are randomly distributed in the space.****

Fig S2. ****Experimental design for the *in vivo* inoculation experiment. All the plants are randomly distribute in each room. All the plants are sampled in each sampling date.****

Analytical solution for the detached-needle assay model fit

Equation 15. Analytical solution for Q in the detached-needle assay, where the solution for *S* is a simple exponential decay, $\boldsymbol{S}\left( \boldsymbol{t} \right)\boldsymbol{=}\boldsymbol{e}^{\boldsymbol{-kPt}}$.

$Q\left( t \right)=\left\{ \begin{matrix} -kPte^{-kPt} \\ \frac{kP}{n-kP}\left( e^{-kPt}-e^{-nt} \right) \end{matrix}\begin{matrix} \mathrm{for} n=kP \\ \mathrm{otherwise} \end{matrix} \right.$ (15)

Detached-needle assay model fit details

Fig S3. Posterior probability distributions of model parameters for the fit of the model with ($\boldsymbol{P=}\boldsymbol{P}_{\boldsymbol{max}}$) to the detached-needle assay data for resistant genotypes.

Simulations to verify stability of on-plant inoculation model fit

Fig S4. Fit of generated data with 30 time points. Solid black line is the model result for the parameter estimates. Thin lines are 400 randomly sampled lines from the full MCMC sampler chain.

Fig S5. Posterior probability distributions of model parameters, for the fit of the model to the generated data with 30 time points. The histograms along the diagonal show the single parameter distributions, the off-diagonal plots show the covariances between parameters.

Fig S6. Fit of generated data with the same number of time points as in the on-plant inoculation. Solid black line is the model result for the parameter estimates. Thin lines are 400 randomly sampled lines from the full MCMC sampler chain.

Fig S7. Posterior probability distributions of model parameters, for the fit of the model to the generated data with the same number of time points as in the on-plant inoculation.

On-plant inoculation model fit details

Fig S8. Posterior probability distributions of model parameters for the model fit to the *detection by isolation* data of the on-plant inoculation.

Fig S9. Posterior probability distributions of model parameters for the model fit to the *detection by qPCR* data of the on-plant inoculation.

Fig S10. Posterior probability distributions of model parameters for the model fit to the *pathogen-to-host DNA ratio* data of the on-plant inoculation.
